# HilD and PhoP independently regulate the expression of *grhD1*, a novel gene required for *Salmonella* Typhimurium invasion of host cells

**DOI:** 10.1038/s41598-018-23068-0

**Published:** 2018-03-19

**Authors:** María M. Banda, Carolina López, Rubiceli Manzo, Gadea Rico-Pérez, Pablo García, Roberto Rosales-Reyes, Miguel A. De la Cruz, Fernando C. Soncini, Francisco García-del Portillo, Víctor H. Bustamante

**Affiliations:** 1 Departamento de Microbiología Molecular, Instituto de Biotecnología, Universidad Nacional Autónoma de México, Cuernavaca, Morelos, 62210 Mexico; 2 Instituto de Biología Molecular y Celular de Rosario, Universidad Nacional de Rosario, Rosario, Argentina; 3 Laboratorio de Patógenos Bacterianos Intracelulares, Centro Nacional de Biotecnología-Consejo Superior de Investigaciones Científicas (CNB-CSIC), Madrid, Spain; 4 Unidad de Investigación en Medicina Experimental, Facultad de Medicina, Universidad Nacional Autónoma de México, Ciudad de México, Mexico; 5 Unidad de Investigación Médica en Enfermedades Infecciosas y Parasitarias, Hospital de Pediatría, Centro Médico Nacional Siglo XXI, Instituto Mexicano del Seguro Social, Ciudad de México, Mexico

## Abstract

When *Salmonella* is grown in the nutrient-rich lysogeny broth (LB), the AraC-like transcriptional regulator HilD positively controls the expression of genes required for *Salmonella* invasion of host cells, such as the *Salmonella* pathogenicity island 1 (SPI-1) genes. However, in minimal media, the two-component system PhoP/Q activates the expression of genes necessary for *Salmonella* replication inside host cells, such as the SPI-2 genes. Recently, we found that the *SL1344_1872* hypothetical gene, located in a *S*. Typhimurium genomic island, is co-expressed with the SPI-1 genes. In this study we demonstrate that HilD induces indirectly the expression of *SL1344_1872* when *S*. Typhimurium is grown in LB; therefore, we named *SL1344_1872* as *grhD1* for gene regulated by HilD. Furthermore, we found that PhoP positively controls the expression of *grhD1*, independently of HilD, when *S*. Typhimurium is grown in LB or N-minimal medium. Moreover, we demonstrate that the *grhD1* gene is required for the invasion of *S*. Typhimurium into epithelial cells, macrophages and fibroblasts, as well as for the intestinal inflammatory response caused by *S*. Typhimurium in mice. Thus, our results reveal a novel virulence factor of *Salmonella*, whose expression is positively and independently controlled by the HilD and PhoP transcriptional regulators.

## Introduction

The acquisition of DNA fragments by horizontal transfer events has played a major role in the evolution of pathogenic bacteria. The acquired DNA may encode different factors that confer the ability to survive and replicate in distinct biological niches within an animal or human host, which leads to bacterial infection and disease^1,2^. To take advantage of the information contained in the acquired DNA, bacteria adapt regulatory mechanisms that allow the expression of the gained genes in those conditions where it is beneficial^3^.

*Salmonella enterica* is an important pathogen of humans and animals, causing a mild self-limiting gastroenteritis or a severe systemic infection^4^. *Salmonella enterica* serotype Typhimurium (*S*. Typhimurium) is a major cause of gastroenteritis in humans and several animals; but can also produce a systemic infection in laboratory mice, similar to the typhoid fever produced by *S*. Typhi in humans^4,5^. Therefore, *S*. Typhimurium is widely used as a model in infections to mice, cattle or eukaryotic cell cultures, to investigate the molecular mechanisms governing *Salmonella* virulence. Most of the virulence genes of *Salmonella* are grouped in acquired genomic regions called *Salmonella* pathogenicity islands (SPIs)^6–8^. SPI-1 and SPI-2 are major determinants for the *Salmonella* intestinal and systemic infection, respectively^8^. SPI-1 is present in the two *Salmonella* species, *S*. *enterica* and *S*. *bongori*, whereas SPI-2 is only conserved in the *S*. *enterica* species, suggesting that SPI-1 was acquired before SPI-2 during *Salmonella* evolution^7,9^. SPI-1 and SPI-2 both encode type III secretion systems (T3SSs), their cognate effector proteins, chaperones and transcriptional regulators controlling the expression of the respective genes within each island^8,10^. During initial infection, *Salmonella* invades host intestinal epithelium using the SPI-1-encoded T3SS (T3SS-1) and cognate effector proteins, which leads to gastroenteritis; by contrast, the SPI-2-encoded T3SS (T3SS-2) and cognate effector proteins provide to *Salmonella* the ability to survive and replicate inside epithelial cells and macrophages; within a membrane-bound compartment called *Salmonella*-containing vacuole (SCV), which leads to the systemic disease^4,8^. The SPI-2 genes also mediate a *Salmonella* non-proliferative stage inside phagocytes and non-phagocytic cells^11,12^ and contribute to the development of the intestinal inflammatory response^13–15^.

The SPI-1 and SPI-2 genes are expressed in different *in vivo* niches; the SPI-1 genes are activated when *Salmonella* is in the intestinal lumen and also in the cytosol of epithelial cells^16,17^; whereas the SPI-2 genes are activated within the SCV of host cells, such as macrophages, epithelial cells and fibroblasts^12,16,18–20^. The SPI-2 genes are also expressed in the intestinal lumen^21^, in the lamina propria or in the underlying mucosa^17^. *In vitro*, the SPI-1 genes are induced when *Salmonella* is grown at early stationary phase in the nutrient-rich lysogeny broth (LB)^22–24^; in contrast, the SPI-2 genes are induced when *Salmonella* is grown at late stationary phase in nutrient-rich media, as well as in minimal media containing low concentrations of phosphate, calcium and magnesium^19,23–25^.

The expression of the SPI-1 genes is controlled by the HilD, InvF and HilA regulators encoded in SPI-1, in a cascade fashion. HilD, an AraC-like transcriptional regulator, induces the expression of HilA, a regulator with an OmpR-ToxR-like DNA binding domain, which in turn activates the expression of InvF, another AraC-like regulator^26–31^. HilA and InvF activate the expression of the SPI-1 genes encoding the T3SS-1 components and effector proteins, respectively^8^. HilD also induces the expression of HilA through a feed-forward regulatory loop that it forms with HilC and RtsA^30,32^, which are AraC-like regulators that bind the DNA sites recognized by HilD^33,34^; HilC and RtsA are encoded within and outside SPI-1, respectively^8^. Furthermore, HilD induces, directly or through HilA, InvF or several other regulators, the expression of many horizontally acquired virulence genes located in different islands, as well as ancestral genes including those for flagella biosynthesis and chemotaxis^8,23,35–43^. Interestingly, HilD is involved in the expression of the *ssrAB* operon encoding the SsrA/B two-component system, the central positive regulator for the SPI-2 genes, but only when *Salmonella* is grown in LB^23,44^. When *Salmonella* is grown in minimal media, the expression of the *ssrAB* operon, and thus the SPI-2 genes, is induced by other regulators such as the MarR-like regulator SlyA and the two-component systems OmpR/EnvZ and PhoP/PhoQ, independently of HilD^8,45^.

The PhoP/PhoQ two-component system is formed by the sensor kinase protein PhoQ and its cognate response regulator PhoP^46–48^. In response to environmental signals such as acidic pH, low concentration of magnesium and antimicrobial peptides, PhoQ autophosphorylates and then phosphorylates PhoP, which binds to target genes^48–53^. Orthologous of PhoP/PhoQ are present in several bacteria, controlling the expression of genes for different cellular functions, including virulence, Mg^2+^ homeostasis, modification of lipopolysaccharides and resistance to antimicrobial peptides and acidic pH^19,48,54–59^. PhoP directly or indirectly regulates the expression of ∼9% of the *S*. Typhimurium genome, including the SPI-2 genes, thus having a fundamental role in phisiology and virulence^19,41,60–62^.

In this study, we show that the transcriptional regulator HilD indirectly induces the expression of the *SL1344_1872* hypothetical gene, when *S*. Typhimurium is grown in LB. Furthermore, we demonstrate that *SL1344_1872*, here named as *grhD1*, ‘gene regulated by HilD’, is required for the invasion of *S*. Typhimurium into host cells and for the intestinal inflammatory response caused by *S*. Typhimurium in mice. In addition, we found that the response regulator PhoP also positively regulates the expression of *grhD1*, directly and independently of HilD, in response to different growth conditions. Therefore, our results from this study reveal a novel *Salmonella* virulence factor, GrhD1, whose expression is controlled by two major transcriptional regulators of *Salmonella* pathogenicity, HilD and PhoP.

## Results

### HilD positively regulates the expression of the *SL1344_1872* (*grhD1*) gene

**Figure 1:**
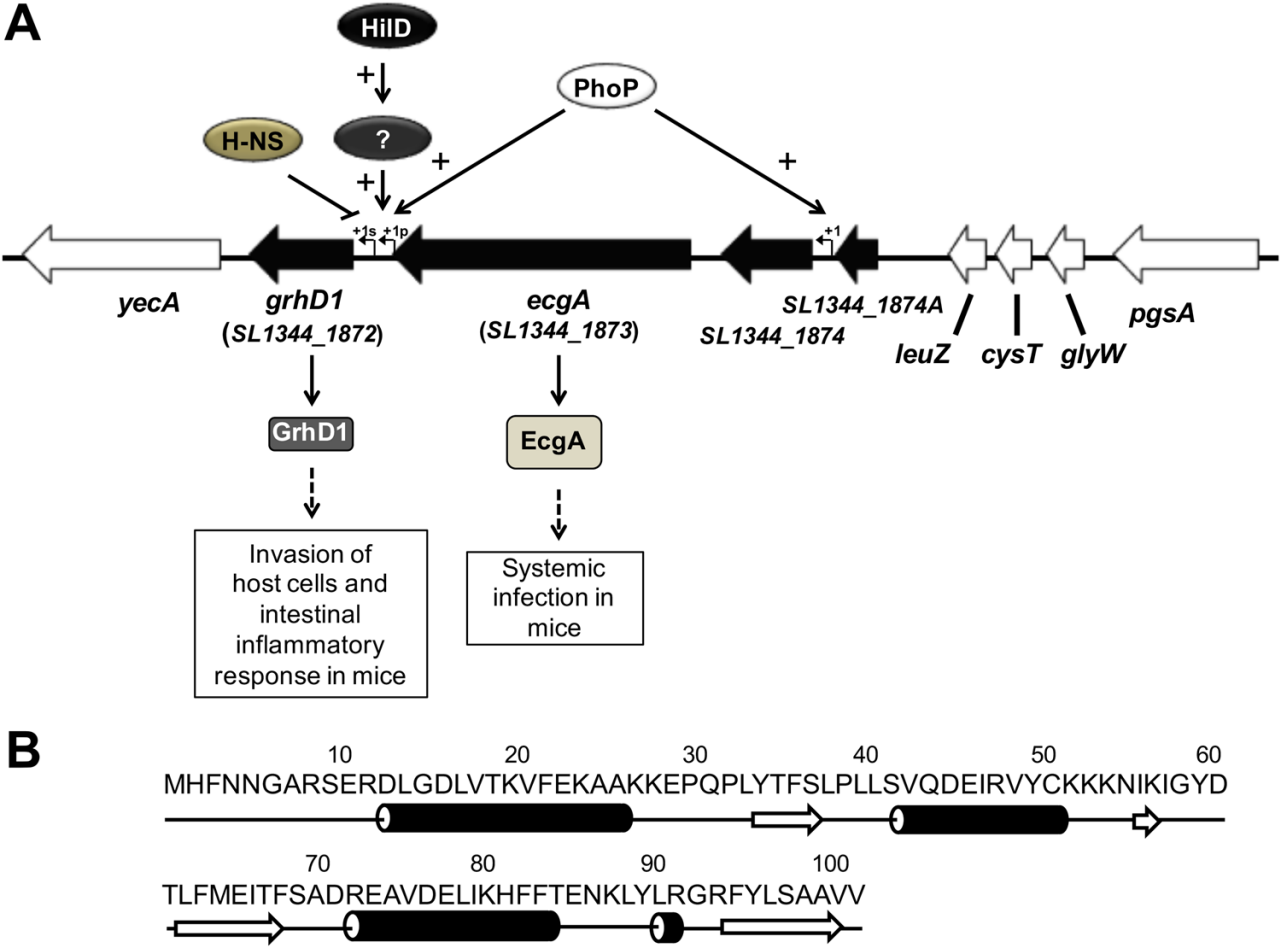
Genetic context, regulatory model and role in virulence of the *grhD1* (*SL1344_1872*) gene of *S*. Typhimurium, as well as sequence and predictive secondary structure of its product. (**A**) Schematic representation of the *S*. Typhimurium SL1344 genome region harbouring *grhD1*. Arrows indicate coding sequences and lines represent intergenic regions. Black arrows indicate all the genes that are located in the genomic island containing *grhD1*. Bent arrows represent the primary (+1p) and secondary (+1s) transcription start sites reported in a previous study^24^. The virulence role for GrhD1 and EcgA, as well as the regulation by HilD, PhoP and H-NS, are also indicated. (**B**) Amino acid sequence and prediction of the secondary structure prediction of GrhD1. Arrows and cylinders indicate predicted β-strands and α-helices, respectively.

In a previous study, we identified a set of novel genes that are co-expressed with the SPI-1 genes in *S*. Typhimurium, by an *in silico* global expression analysis^42^. The characterization of some of these genes revealed a strong link between the co-expression with SPI-1 and the regulation by HilD. One uncharacterized gene co-expressed with SPI-1 is *SL1344_1872*, which is located in a *S*. Typhimurium acquired genomic island flanked by the *yecA* gene of unknown function and the tRNA-encoding *leuZ* gene (Fig. 1A). This island contains two additional genes, *SL1344_1873* (*ecgA*) and *SL1344_1874*, as well as the pseudogene *SL1344_1874A*; *SL1344_1873* and *SL1344_1874* encode a peptidoglycan enzyme (EcgA) with L-endopeptidase activity, involved in *S*. Typhimurium virulence, and a hypothetical membrane protein, respectively (Fig. 1A)^63^. The *SL1344_1872* gene encodes a hypothetical protein of 101 amino acids, predicted to form four α-helices and four β-strands (Fig. 1B). SL1344_1872 has no orthologs in other bacteria and does not present any conserved domain. Recent transcriptomic analysis supports that HilD positively regulates the expression of *SL1344_1872*^41^.

**Figure 2:**
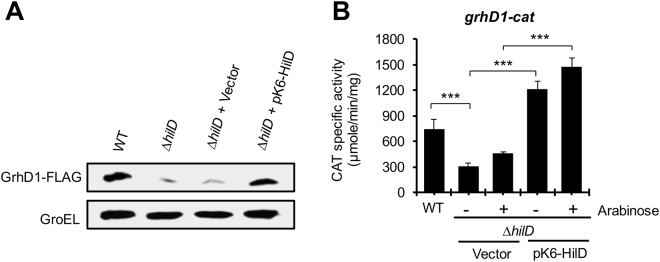
HilD positively regulates the expression of *grhD1* (*SL1344_1872*). (**A**) Expression of GrhD1-FLAG in the WT *S*. Typhimurium SL1344 strain and its derivative ∆*hilD* mutant containing or not the pK6-HilD plasmid, which expresses HilD from an arabinose-inducible promoter, or containing the pMPM-K6Ω vector. The expression of GrhD1-FLAG was analyzed from samples of bacterial cultures grown in LB at 37 °C by Western blotting, using monoclonal anti-FLAG antibodies. The expression of GroEL was also determined using polyclonal anti-GroEL antibodies, as a loading control. (**B**) Expression of the *grhD1-cat* transcriptional fusion contained in the pgrhD1-cat plasmid was determined in the WT *S*. Typhimurium strain and its derivative ∆*hilD* mutant carrying or not pK6-HilD or pMPM-K6Ω. Expression of HilD from pK6-HilD was activated by adding 0.001% L-arabinose to the medium. CAT specific activity was determined from samples of bacterial cultures grown in LB at 37 °C. Data are the average of three independent experiments done in duplicate. Bars represent the standard deviations. Statistically different values are indicated (***p < 0.001).

To determine whether *SL1344_1872* indeed codes for a protein and to confirm whether its expression is controlled by HilD, the *SL1344_1872* chromosomal gene was tagged in the wild-type (WT) *S*. Typhimurium SL1344 strain and its isogenic ∆*hilD* mutant, with the sequence encoding a 3XFLAG epitope. Total protein extracts were obtained from culture samples of these strains grown in LB at 37 °C, conditions that induce the expression of the SPI-1 genes, which were analyzed by Western blotting using anti-FLAG antibodies. An expression signal was detected in the WT strain, with the expected size for the SL1344_1872-FLAG protein (Fig. 2A). The expression of SL1344_1872-FLAG was drastically reduced in the ∆*hilD* mutant; the pK6-HilD plasmid expressing HilD restored the expression of SL1344_1872-FLAG in the ∆*hilD* mutant to WT levels (Fig. 2A). To investigate whether HilD regulates *SL1344_1872* at transcriptional level, a transcriptional fusion of the intergenic region upstream of *SL1344_1872* to the *cat* reporter gene was constructed in the pKK232-8 plasmid. The chloramphenicol acetyl transferase (CAT)-specific activity from this fusion was determined in the WT *S*. Typhimurium strain and its isogenic ∆*hilD* mutant, grown in LB. The activity of the *SL1344_1872-cat* fusion showed a 2-fold decrease in the ∆*hilD* mutant with respect to the WT strain, and was induced 3-fold in the ∆*hilD* mutant by the expression of HilD from the pK6-HilD plasmid (Fig. 2B). Together, these results demonstrate that HilD positively regulates the expression of *SL1344_1872*, herein named *grhD1* for gene regulated by HilD.

We next investigated whether the control of *grhD1* expression by HilD is direct or indirect. Hence, electrophoretic mobility shift assays (EMSAs) were performed using affinity-purified maltose-binding protein (MBP)-HilD and the DNA fragment carrying the intergenic region upstream of *grhD1*. DNA fragments containing the regulatory regions of *hilA* or *sigD* were also tested as positive and negative controls, respectively. As expected, MBP-HilD specifically bound the DNA fragment of *hilA*, at concentrations of 0.5 to 1 µM; in contrast, at the same concentrations it did not shift the DNA fragment of *grhD1*, or that of the negative control, *sigD* (Fig. S1A and B). These results support that HilD regulates *grhD1* indirectly; alternatively, an additional factor could be required for HilD binding on *grhD1*.

*Escherichia coli* K-12 lacks *hilD*, *hilA* and *grhD1*, as well as around 1400 other genes present in *S*. Typhimurium. The expression of genes known to be directly controlled by HiD, such as *hilA*, can be induced in *E. coli* K-12 when HilD is present^42^. Therefore, to further test if HilD regulates *grhD1* indirectly, the activity of the *grhD1-cat* fusion was determined in the *E. coli* MC4100 strain carrying the pK6-HilD plasmid, grown in LB. As a positive control, a *hilA-cat* transcriptional fusion was also examined. As expected, the activity of both *grhD1-cat* and *hilA-cat* fusions was decreased in *E. coli* MC4100 with respect to the WT *S*. Typhimurium strain (Fig. S1C and D). Expression of HilD from the pK6-HilD plasmid activated the *hilA-cat* fusion, but not the *grhD1-cat* fusion, in *E. coli* MC4100 (Fig. S1C and D), indicating that an additional *Salmonella* factor is required for the HilD-mediated expression of *grhD1*.

HilD induces the expression of several transcriptional regulators, including HilC, HilA and InvF, encoded in SPI-1, as well as RtsA, SsrB and FlhDC, encoded outside SPI-1^8,23,31,38,40^. To investigate whether HilD regulates *grhD1* through any of these regulators, the expression of the *grhD1-cat* fusion was determined in the WT *S*. Typhimurium strain and its derivative ∆SPI-1 ∆*rtsA* mutant, as well as in ∆*ssrB*, ∆*flhDC*, ∆*hilA*, and ∆*invF* mutants, grown in LB. As expected, the *grhD1-cat* fusion presented a 2-fold-reduced expression in the ∆SPI-1 ∆*rtsA* mutant, since it lacks HilD; nevertheless, its expression was restored to WT levels in the presence of the pK6-HilD plasmid (Fig. S2A). On the other hand, the *grhD1-cat* fusion showed similar expression levels in the WT strain and its isogenic ∆*ssrB*, ∆*flhDC*, ∆*hilA*, and ∆*invF* mutants (Fig. S2B). These results indicate that the expression of *grhD1* induced by HilD does not require any other regulator encoded in SPI-1 (HilC, HilA, InvF, SprB), neither RtsA, SsrB or FlhDC, in the growth condition tested.

Collectively, these results show that HilD indirectly induces the expression of *grhD1*, through a yet-unknown regulator controlled by HilD, found in *S*. Typhimurium but not in *E. coli* MC4100.

### PhoP positively regulates the expression of *grhD1*

**Figure 3:**
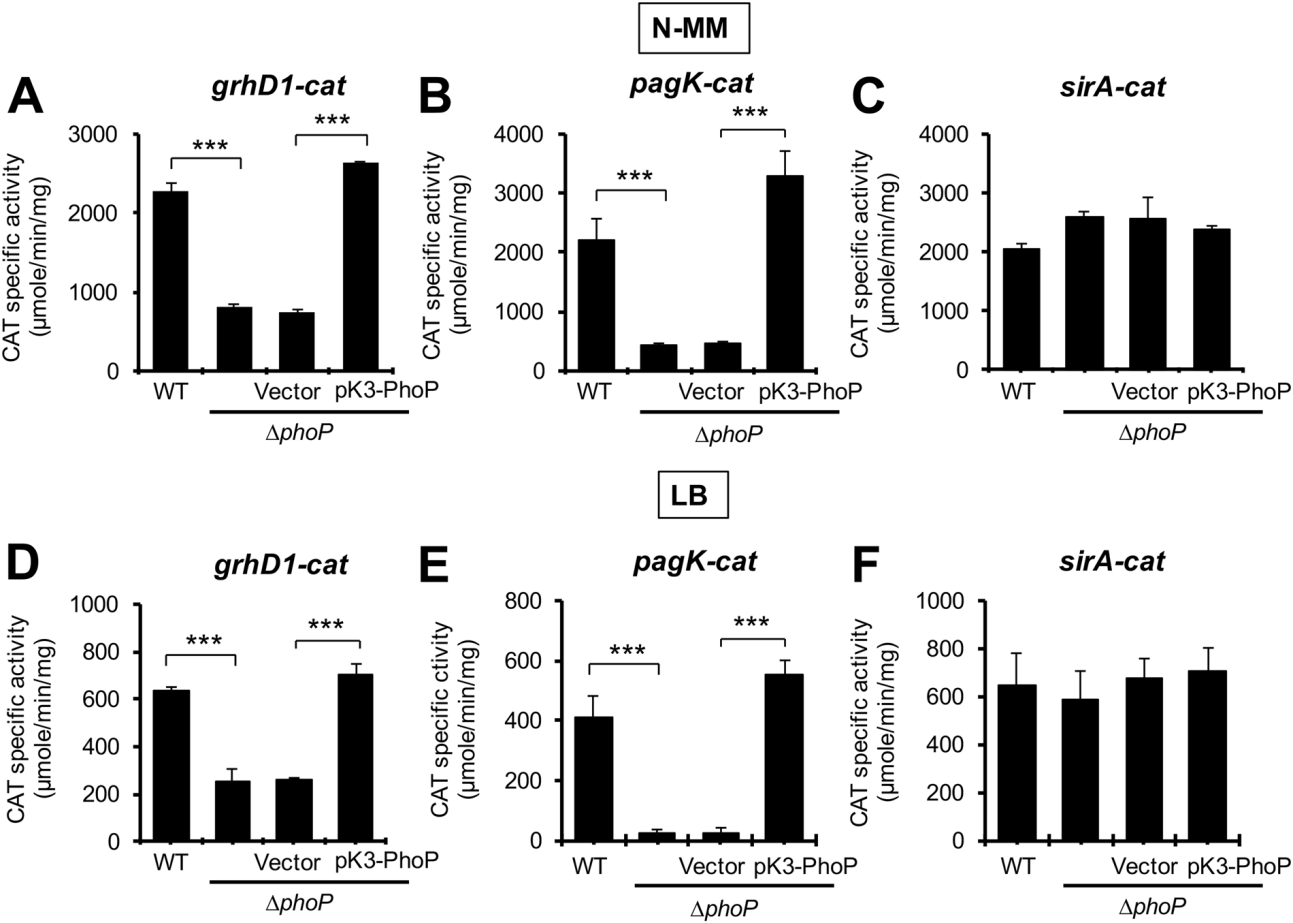
PhoP actives the expression of *grhD1*. Expression of the *grhD1-cat* (**A,D**), *pagK-cat* (**B,E**) and *sirA-cat* (**C,F**) transcriptional fusions contained in the pgrhD1-cat, ppagK-cat and psirA-cat plasmids, respectively, was determined in the WT *S*. Typhimurium strain and its derivative ∆*phoP* mutant containing or not the pK3-PhoP plasmid or the pMPM-K3 vector, grown in N-MM (**A**–**C**) or LB (**D–F**) at 37 °C. pK3-PhoP constitutively expresses PhoP from a *lac* derivative promoter. Data are the average of three independent experiments done in duplicate. Bars represent the standard deviations. Statistically different values are indicated (***p < 0.001).

Recently, we reported that the response regulator PhoP positively and directly controls the expression of the operon containing the *ecgA* and *SL1344_1874* genes, which is located close to *grhD1*, in the same *S*. Typhimurium genomic island (Fig. 1A)^63^. Therefore, we thought that PhoP could also be involved in the expression of *grhD1*, which is supported by recent transcriptomic analyses^41^. In order to determine this, we examined the activity of the *grhD1-cat* transcriptional fusion in the WT *S*. Typhimurium strain and its derivative ∆*phoP* mutant, grown in LB or N-minimal medium (N-MM). PhoP is known to be active when *S*. Typhimurium is grown in the nutrient-rich LB or in minimal media containing low concentrations of magnesium^63^. As positive and negative controls, the activity of transcriptional fusions to *cat* reporter of *pagK*, a gene positively regulated by PhoP, and *sirA*, a gene not regulated by PhoP, was also tested. The activity of the *grhD1-cat*, *pagK-cat* and *sirA-cat* fusions was higher in N-MM than in LB; however, the expression pattern for each fusion in the different genetic backgrounds tested was similar in both growth conditions (Fig. 3). The activity of the *grhD1-cat* and *pagK-cat* fusions was reduced in the ∆*phoP* mutant, with respect to the WT strain; expression of PhoP from pK3-PhoP recovered the activity of both fusions in the ∆*phoP* mutant to WT levels (Fig. 3A,B,D and E). In contrast, the activity of the *sirA-cat* fusion was not affected by the absence or overexpression of PhoP (Fig. 3C and F). To further support these results, we monitored the expression of GrhD1-FLAG in the WT strain and the ∆*phoP* mutant, grown in LB, intracellular salt medium (ISM) or acidified PCN (phosphate-carbon-nitrogen) medium. In all the conditions tested, the expression of GrhD1-FLAG was severely reduced in the ∆*phoP* mutant with respect to the WT strain (Fig. S3A and B). As expected, the presence of the pK3-PhoP plasmid restored the expression of GrhD1-FLAG in the ∆*phoP* mutant to WT levels (Fig. S3B). These results indicate that PhoP positively regulates the expression of *grhD1* in *S*. Typhimurium growing in LB or minimal media.

**Figure 4:**
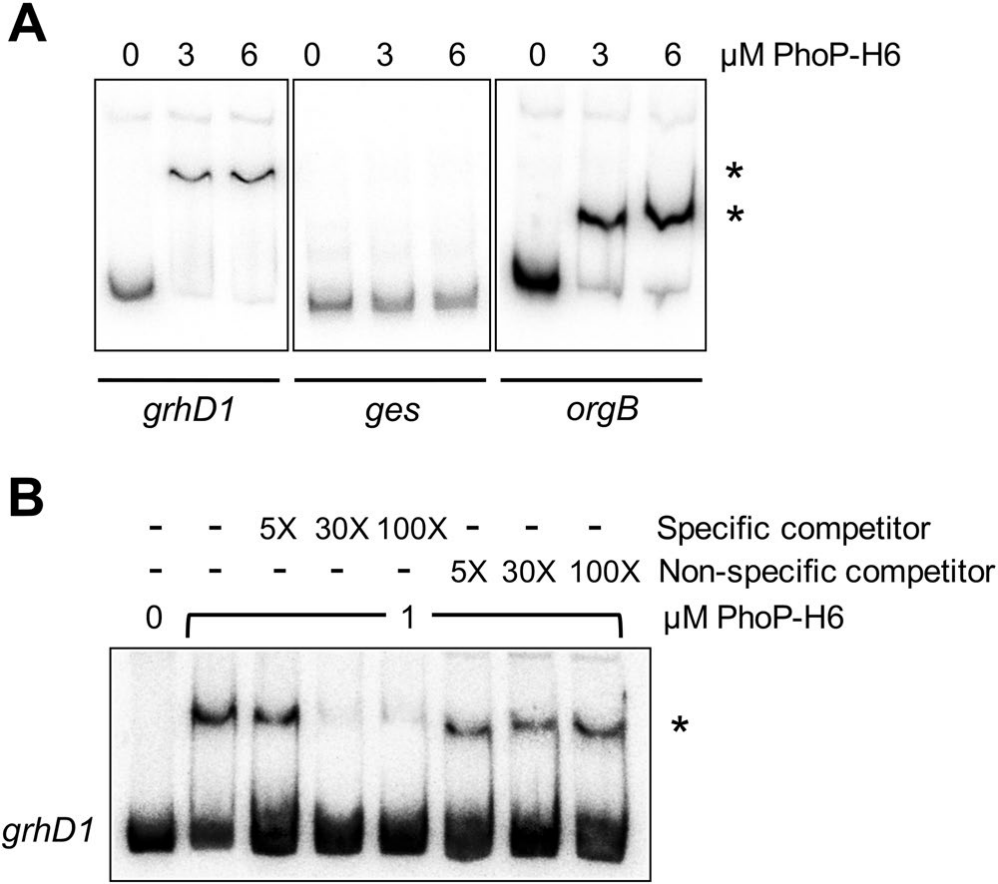
PhoP specifically binds to the *grhD1* regulatory region. EMSAs with PhoP-H6 and DNA fragments containing the regulatory regions of the *grhD1*, *ges* or *orgB* genes (**A**). ^32^P-5′-end-labelled DNA fragments of the respective gene were incubated with increasing concentrations of purified and phosphorylated PhoP-H6 (0, 3 and 6 µM). The *ges* and *orgB* genes were used as negative and positive controls, respectively. PhoP binding to *grhD1* was further tested by competitive EMSAs (**B**). The ^32^P-5′-end-labelled DNA fragment of *grhD1* was incubate with 1 µM of purified and phosphorylated PhoP-H6 in the absence or presence of 5-, 30- and 100-fold excess of unlabelled specific (*grhD1*) or non-specific (*nucA*) competitors. The DNA-protein complexes, which are indicated by an asterisk, were resolved in a nondenaturing 8% Tris-borate-EDTA-polyacrylamide gel. After electrophoresis, the gel was dried and analyzed in a Typhoon FLA 7000 IP laser scanner.

To determine whether PhoP regulates *grhD1* directly or indirectly, we performed EMSAs with phosphorylated affinity-purified PhoP-6XHis (PhoP-H6) fusion protein and a labelled DNA fragment containing the regulatory region of *grhD1*. DNA fragments carrying the regulatory region of the *orgB*^56^ or *ges*^64^ genes were also tested as positive and negative controls, respectively. PhoP-H6 shifted the *grhD1* and *orgB* fragments starting at a concentration of 3 µM; in contrast, the *ges* fragment was not shifted even at a concentration of 6 µM (Figs 4A and S4). Specific binding of PhoP-H6 to *grhD1* was confirmed by competitive EMSAs (Fig. 4B). Together with the results from expression analyses, these binding assays indicate that PhoP directly regulates the expression of the *grhD1* gene.

### HilD and PhoP independently control the expression of *grhD1*

**Figure 5:**
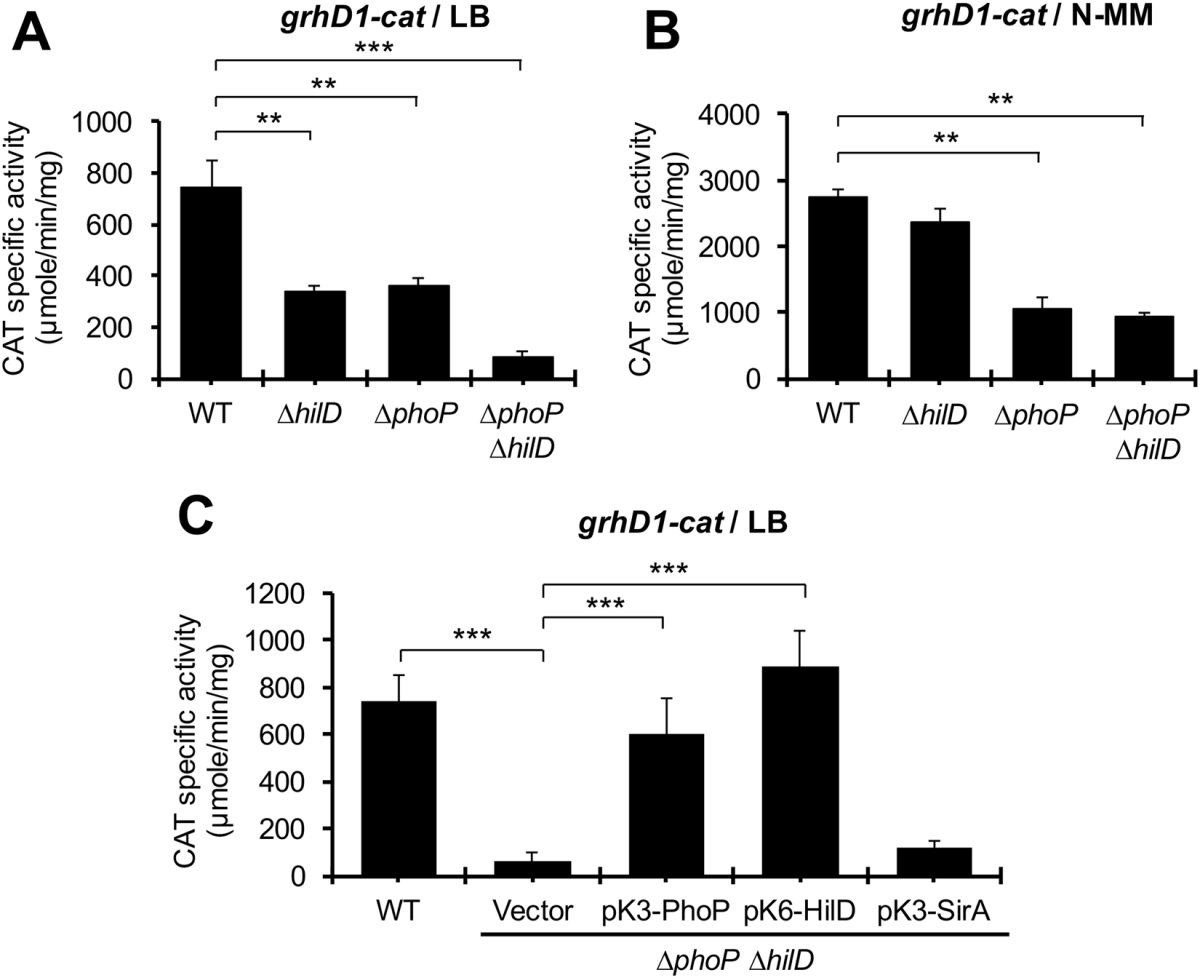
HilD and PhoP induce the expression of *grhD1* independently. Expression of the *grhD1-cat* transcriptional fusion contained in the pgrhD1-cat plasmid was determined in the WT *S*. Typhimurium and its derivative ∆*hilD*, ∆*phoP* and ∆*phoP* ∆*hilD* mutants grown in LB (**A**) or N-MM (**B**) at 37 °C, as well as in the WT S. Typhimurium strain and its isogenic ∆*phoP* ∆*hilD* mutant carrying or not the pMPM-K3 vector or the pK3-PhoP, pK6-HilD and pK3-SirA plasmids, grown in LB at 37 °C (**C**). Expression of HilD from pK6-HilD was induced by adding 0.001% L-arabinose to the medium at the beginning of the bacterial cultures. pK3-PhoP and pK3-SirA constitutively express PhoP and SirA, respectively. Data are the average of three independent experiments done in duplicate. Bars represent the standard deviations. Statistically different values are indicated (**p < 0.01; ***p < 0.001).

Our results indicate that both HilD and PhoP positively control the expression of *grhD1*, which could act independently of each other or one through the other; for instance, since HilD regulates *grhD1* indirectly, it could act through PhoP. To investigate this, firstly, the expression of the *grhD1-cat* fusion was compared in the WT *S*. Typhimurium strain and its isogenic ∆*hilD*, ∆*phoP* and ∆*phoP* ∆*hilD* mutants, grown in LB or N-MM. In LB, the *grhD1-cat* fusion showed a similar 2-fold reduction of its activity in the ∆*hilD* and ∆*phoP* mutants with respect to the WT strain, whereas in the ∆*phoP* ∆*hilD* double mutant its activity was even 4-fold lower than in the single mutants (Fig. 5A), indicating that HilD and PhoP have an additive effect on *grhD1*. In contrast, in N-MM, the activity of the *grhD1-cat* fusion was not affected in the ∆*hilD* mutant and presented a similar 2.5-fold decrease in the ∆*phoP* and ∆*phoP* ∆*hilD* mutants (Fig. 5B), showing that PhoP regulate *grhD1* independently of HilD in these growth conditions. Then, we determined the activity of the *grhD1-cat* fusion in the ∆*phoP* ∆*hilD* double mutant expressing PhoP, HilD and SirA from the pK3-PhoP, pK6-HilD or pK3-SirA plasmids, respectively, grown in LB. SirA is a transcriptional regulator expected to be not involved in the expression of *grhD1*. Expression of PhoP or HilD, but not SirA, induced the activity of the *grhD1-cat* fusion in the ∆*phoP* ∆*hilD* mutant (Fig. 5C). Together, these results show that PhoP and HilD regulate *grhD1* independently of each other; interestingly, the overexpression of one of these regulators compensates the absence of the other for *grhD1* expression.

**Figure 6:**
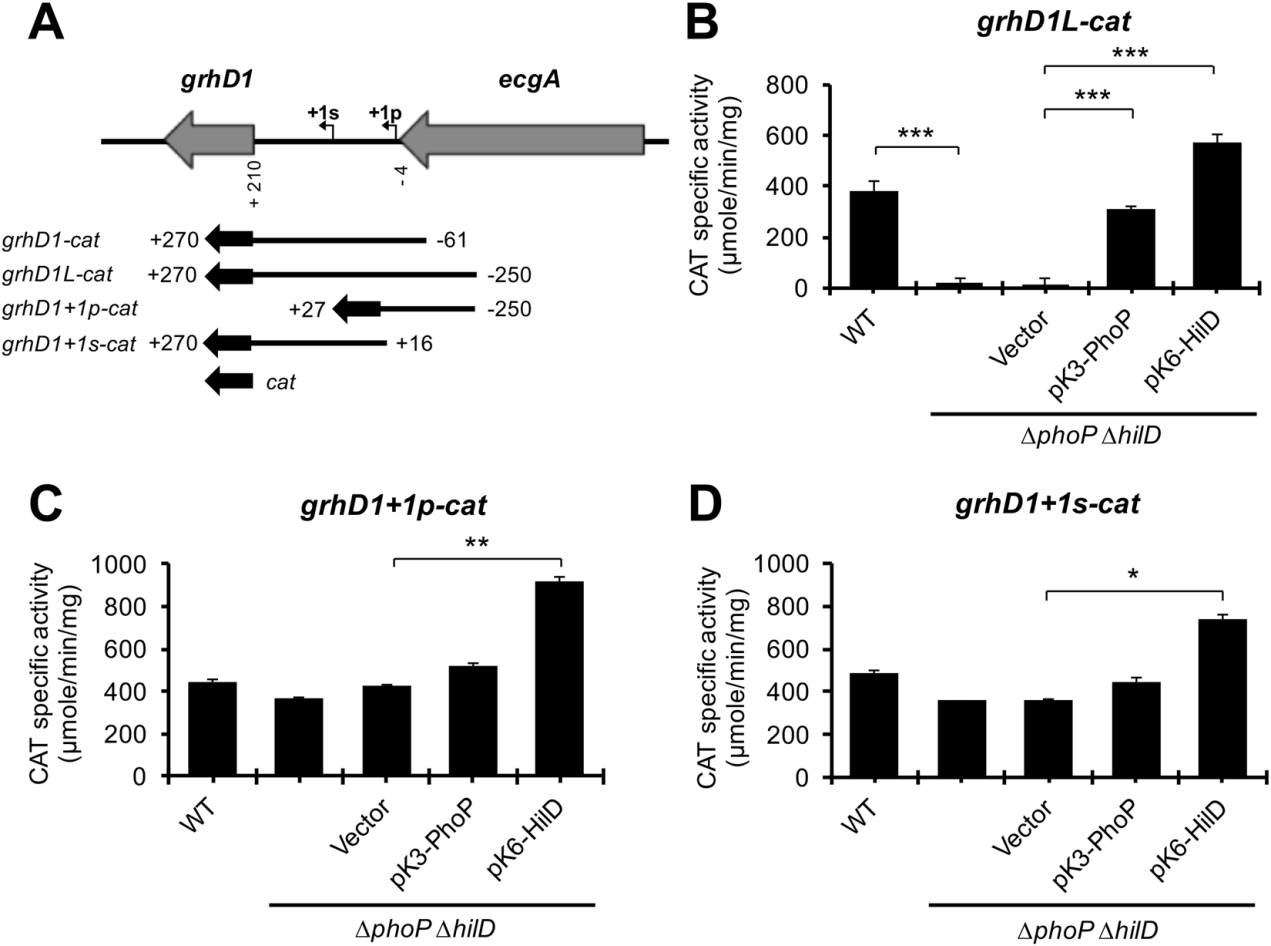
Two different promoters regulated by HilD and PhoP induce the expression of *grhD1*. Schematic representation of the intergenic region upstream *grhD1* (**A**). The primary (+1p) and secondary (+1s) transcription start sites of *grhD1*, previously reported^24^, are indicated by a bent arrow. The DNA fragments carried by the *grhD1-cat*, *grhD1L-cat*, *grhD1* + *1p-cat* and *grhD1* + *1s-cat* transcriptional fusions, are shown; positions are indicated with respect to the primary transcriptional start site of *grhD1*. Expression of the *grhD1L-cat* (**B**), *grhD1* + *1p-cat* (**C**), and *grhD1* + *1s-cat* (**D**) transcriptional fusions contained in the pgrhD1L-cat, pgrhD1 + 1p-cat and pgrhD1 + 1s-cat plasmids, respectively, was tested in the WT *S*. Typhimurium strain and its isogenic ∆*phoP* ∆*hilD* mutant carrying or not the pK3-PhoP or pK6-HilD plasmids, or the pMPM-K3 vector. CAT specific activity was determined from samples of bacterial cultures grown in LB at 37 °C. Expression of HilD from pK6-HilD was induced by adding 0.001% L-arabinose to the medium at the beginning of the bacterial cultures. pK3-PhoP constitutively expresses PhoP. Data are the average of three independent experiments done in duplicate. Bars represent the standard deviations. Statistically different values are indicated (*p < 0.05; **p < 0.01; ***p < 0.001).

Previous RNA-sequencing analyses revealed a primary and a secondary transcriptional start site (TSS) in the intergenic region upstream of *grhD1*, located at 230 and 148 bp from the start codon of *grhD1*, respectively (Fig. 6A)^24^. The *grhD1-cat* fusion tested in the experiments described above carries a DNA region containing these two TSSs. Therefore, to define whether the expression of *grhD1* is sustained by two promoters and in that case, whether HilD and PhoP each affects one or both promoters, we constructed three additional *grhD1-cat* transcriptional fusions. The *grhD1L-cat* fusion carries an extended 3′ *grhD1* upstream region with respect to that contained in the initial assessed *grhD1-cat* fusion, whereas the *grhD1* + *1p-cat* and *grhD1* + *1s-cat* fusions carry segments of the *grhD1* upstream region containing only the primary or secondary TSS, respectively (Fig. 6A). The activity of these new constructed fusions was monitored in the WT *S*. Typhimurium strain and its isogenic ∆*phoP* ∆*hilD* double mutant, grown in LB. The *grhD1L-cat, grhD1* + *1p-cat* and *grhD1* + *1s-cat* fusions were similarly expressed in the WT strain (Fig. 6B–D), indicating that two independent promoters sustain the expression of *grhD1* in these growth conditions. The *grhD1L-cat* fusion, carrying both promoters, showed a 17-fold-reduced activity in the Δ*phoP* Δ*hilD* mutant, which was restored to WT levels or even higher by the expression of PhoP or HilD from the pK3-PhoP or pK6-HilD plasmids, respectively (Fig. 6B). Thus, these results, together with those from Fig. 5C, support that HilD and PhoP independently induce the transcription of both *grhD1* promoters. Surprisingly, the activity of the *grhD1* + *1p-cat* and *grhD1* + *1s-cat* fusions was not significantly affected in the ∆*phoP* ∆*hilD* mutant containing or not the pK3-PhoP plasmid, with respect to the WT strain; only the presence of pK6-HilD further increased the activity of these fusions (Fig. 6C and D). These results suggest that proper control of *grhD1* expression requires negative regulatory sequences located around the promoters; in the absence of these sequences the expression of *grhD1* becomes independent of HilD and PhoP.

### H-NS represses the expression of *grhD1*

The histone-like protein H-NS works as a global transcriptional regulator that silences the expression of genes acquired by *Salmonella*^65,66^. We investigated whether the negative regulatory sequences on *grhD1* could mediate repression by H-NS. Given that the *hns* null mutation generates severe growth defects in *Salmonella*^66^, we analyzed the effect of H-NS on *grhD1* by overexpressing the H-NS^G113D^ mutant, which does not have DNA-binding activity but still forms heterodimers with WT H-NS monomers^67^ and thus acts as a dominant negative mutant^68^. The activity of the *grhD1-cat* fusion was tested in the ∆*hilD* and ∆*phoP* mutants containing the pT6-HNS-G113D plasmid expressing H-NS^G113D^, as well as in the WT strain. The expression of H-NS^G113D^ induced the activity of the *grhD1-cat* fusion in the ∆*hilD* mutant, but not in the ∆*phoP* mutant (Fig. S5A). These results indicate that with the inactivation of H-NS the expression of *grhD1* becomes independent of HilD, thus revealing that H-NS represses *grhD1*.

To determine whether H-NS regulates *grhD1* directly or indirectly, we performed EMSAs with the affinity-purified H-NS-3XFLAG-6XHis (H-NS-FH) protein and a fragment containing the *grhD1* regulatory region. DNA fragments containing the regulatory regions of *ssrAB* or *sigD* were also tested as positive and negative controls, respectively. H-NS-FH bound the DNA fragments of *grhD1* and *ssrAB*, starting at a concentration of 0.5 and 0.25 µM, respectively; as expected, it did not bind the negative control *sigD* (Fig. S5B and C). Together with the expression analyses, these binding assays demonstrate that H-NS directly represses the expression of *grhD1*.

### GrhD1 is required for invasion of *S*. Typhimurium into host cells

**Figure 7:**
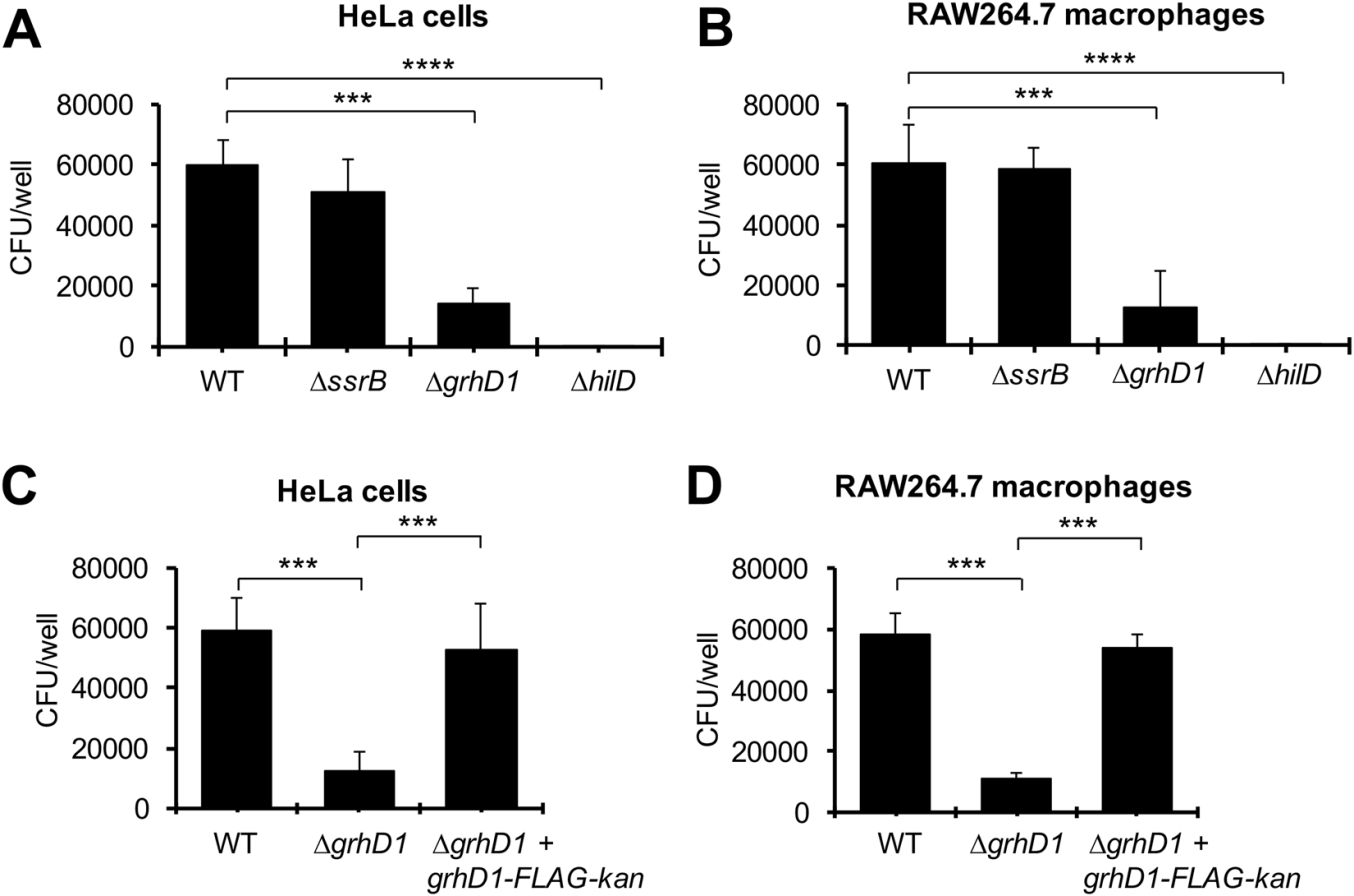
GrhD is required for invasion of *S*. Typhimurium into HeLa cells and macrophages. Epithelial HeLa cells (**A**) and murine RAW 264.7 macrophages (**B**) were infected with the WT *S*. Typhimurium strain or its isogenic ∆*ssrB*, ∆*grhD1* and ∆*hilD* mutants. Epithelial HeLa cells (**C**) and murine RAW 264.7 macrophages (**D**) were infected with the WT *S*. Typhimurium strain and its isogenic ∆*grhD1* and ∆*grhD1* + *grhD1-FLAG-kan* mutants. Invasion was measured by enumerating the intracellular CFUs at 1 h post-infection, using a gentamicin protection assay. Data are the average of three independent experiments done in triplicate. Bars represent the standard deviations. Statistically different values are indicated (***p < 0.001; ****p < 0.0001).

HilD controls the expression of a high number of genes mainly required for *Salmonella* invasion of host cells^8,40–43^. Therefore, we investigated whether the *grhD1* gene, also regulated by HilD, is involved in this virulence phenotype. Gentamicin protection assays were used to analyze the bacterial invasion of the WT *S*. Typhimurium strain and its derivative ∆*grhD1* mutant into HeLa cells and RAW264.7 mouse macrophages. The ∆*hilD* and ∆*ssrB* mutants were also assessed as positive and negative controls, respectively. The ∆*grhD1* mutant showed a 4-fold reduction in the invasion of both HeLa cells and macrophages in comparison to the WT strain (Fig. 7A and B). As expected, the ∆*ssrB* mutant was not affected in the invasion phenotype and the ∆*hilD* mutant was unable to invade the HeLa cells and macrophages (Fig. 7A and B). The invasion defect of the ∆*grhD1* mutant was also observed in NRK-49F fibroblasts using two different initial infection times (10 and 20 min) (Fig. S6). A centrifugation step can be used in the invasion assays to accelerate the contact between bacteria and eukaryotic cells, which overcomes an invasion deficiency by a motility defect^69^. Even with the centrifugation step the Δ*grhD1* mutant showed a reduced invasion phenotype in HeLa cells, compared to the WT strain (Fig. S7A); in contrast, the Δ*flhDC* mutant, which lacks of motility, greatly recovered its ability to invade HeLa cells with the centrifugation step (Fig. S7B). Next, we sought to complement the invasion phenotype of the ∆*grhD1* mutant with a plasmid expressing GrhD1. For this, we constructed the low-copy number pK3-GrhD1 and pK3-GrhD1-FLAG plasmids, which constitutively express GrhD1 and GrhD1-FLAG proteins, respectively, from a constitutive *lac* promoter. Unexpectedly, the pK3-GrhD1 and pK3-GrhD1-FLAG plasmids further decreased the invasion of the ∆*grhD1* mutant to HeLa cells; moreover, these plasmids also drastically inhibited the invasion of the WT strain (Fig. S8A). To further explore this phenomenon, we monitored by Western blot the expression of the GrhD1-FLAG protein from the *grhD1::3XFLAG* gene located in the chromosome and that carried by the pK3-GrhD1-FLAG plasmid. As shown in Fig. S8B, GrhD1-FLAG reached much higher levels from the pK3-GrhD1-FLAG plasmid that from the chromosomal gene. Together, these results support that both the absence and overexpression of GrhD1 negatively affects the *S*. Typhimurium invasion of host cells independently of motility.

Since a specific concentration of GrhD1 seems to be required for its role in the *S*. Typhimurium invasion of host cells, we decided to complement the ∆*grhD1* mutant by inserting the *grhD1::3XFLAG* allele into the chromosome (Fig. S9). Expression of GrhD1-FLAG was similar in the complemented ∆*grhD1* + *grhD1-FLAG-kan* strain and the WT strain carrying the *grhD1::3XFLAG* allele (Fig. S8C). Accordingly, the complemented ∆*grhD1* + *grhD1-FLAG-kan* strain presented an invasion phenotype similar to that of the WT strain, in both HeLa cells and macrophages (Fig. 7C and D). These results confirm that GrhD1 is an additional *S*. Typhimurium factor required for invasion of host cells.

We also investigated whether *grhD1* have a role during survival/replication of *Salmonella* inside host cells. For this, we infected RAW264.7 macrophages and HeLa cells with the WT *S*. Typhimurium strain and its isogenic ∆*grhD1* mutant, as well as with the ∆*ssrB* mutant, used as a positive control. As expected, the ∆*ssrB* mutant presented an affected replication/survival ability, which was more evident in macrophages that in HeLa cells; in contrast, the replication/survival phenotype of the ∆*grhD1* mutant was very similar to that showed by the WT strain, in both macrophages and HeLa cells (Fig. S10A and B).

In all, these results indicate that *grhD1* is necessary for invasion of *S*. Typhimurium into host cells, but not for its intracellular replication/survival.

### GrhD1 is required for the intestinal inflammatory response induced by *S*. Typhimurium in mice

**Figure 8:**
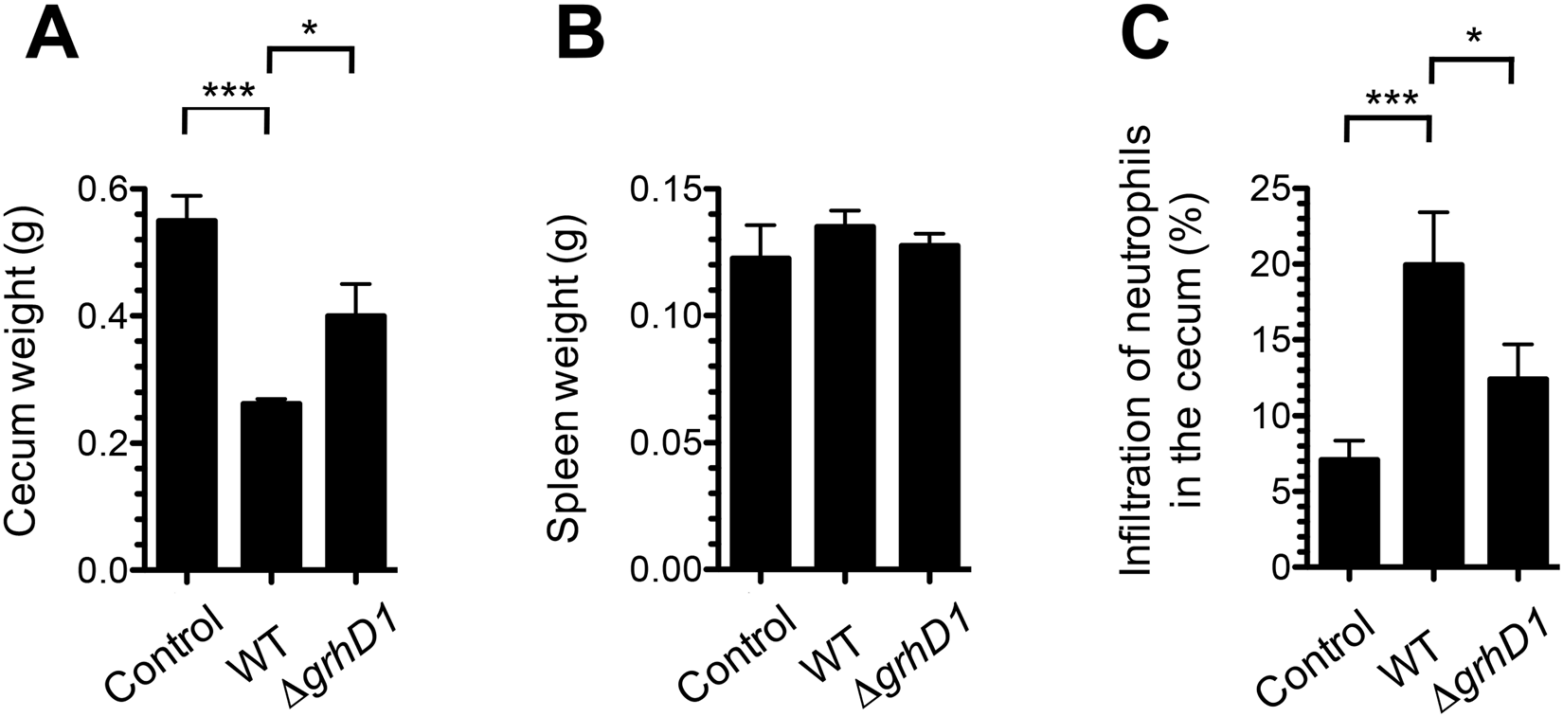
GrhD1 is involved in the intestinal inflammatory response induced by *S*. Typhimurium in mice. Mice pretreated with streptomycin were infected with the WT *S*. Typhimurium strain or the Δ*grhD1* mutant, or treated with sterile 1X PBS (Control). The mice were sacrificed two days post-infection and the total weight (g) of the cecum (**A**) and the spleen (**B**), as well as the infiltration of neutrophils in the cecum content (**C**), was measured for each mouse. Samples of the cecum content were analyzed by microcopy to determine the number of neutrophils with respect to the total eukaryotic cells, which is expressed as a percentage score. Data are the average from four separate animals. Bars represent the standard deviations. Statistically different values are indicated (*p < 0.05; ***p < 0.001).

*Salmonella* invasion of intestinal epithelial cells eventually leads to the development of enterocolitis^8^. To determine whether GrhD1 is necessary for the induction of intestinal inflammation, we analyzed the infection caused by the WT *S*. Typhimurium strain and the ∆*grhD1* mutant in streptomycin-pretreated mice, which is used as a *S*. Typhimurium colitis model^70^. As expected, the WT strain was able to colonize the intestine (cecum and ileum) and the spleen of mice (Fig. S11). Furthermore, the mice infected with the WT strain showed a reduced cecum weight and an increased infiltration of neutrophils in the cecum content (Fig. 8A and C), two hallmarks of the intestinal inflammatory response induced by *S*. Typhimurium in streptomycin-pretreated mice^70^. Interestingly, the ∆*grhD1* mutant colonized the cecum similarly to the WT strain (Fig. S11); however, the mice infected with the ∆*grhD1* mutant showed a significant higher weight of the cecum and a significant lower infiltration of neutrophils in the cecum content, than those infected with the WT strain (Fig. 8A and C). Additionally, the ∆*grhD1* mutant showed a 3-fold reduction in the colonization of the ileum, with respect to the WT strain (Fig. S11). In contrast, the ∆*grhD1* mutant and the WT strain colonized similarly the spleen (Fig. S11) and the mice infected with these strains showed a similar weight of the spleen (Fig. 8B). These results show that GrhD1 is involved in the intestinal infection, particularly in the intestinal inflammatory response induced by *S*. Typhimurium in the streptomycin mouse model.

## Discussion

HilD and PhoP are two major transcriptional regulators controlling the expression of virulence genes in *Salmonella*^8^. Our results reveal one additional virulence gene, *grhD1*, which is regulated by HilD and PhoP. When *S*. Typhimurium proliferates in conditions that favor expression of the SPI-1 and other genes required for the *Salmonella* invasion of host cells, both HilD and PhoP are required for the expression of *grhD1*. We show that HilD and PhoP independently affect two promoters located upstream *grhD1*; PhoP directly and HilD through an additional factor that remains to be identified. Furthermore, our data also show that the histone-like protein H-NS directly represses the expression of *grhD1*; H-NS silences the expression of many other genes acquired by *Salmonella*^65,66^. A model for the regulation of the *grhD1* expression is depicted in Fig. 1A.

HilD and PhoP also positively and independently regulate the expression of the *orgBC* SPI-1 operon, in SPI-1-inducing growth conditions, PhoP directly and HilD through HilA^8,56^. This operon codes for a cytoplasmic protein required for invasion and an effector protein secreted through the T3SS-1, OrgB and OrgC, respectively^71,72^. Opposite regulation mediated by HilD and PhoP on the *hilA* gene, encoding a master regulator for the SPI-1 genes, has also been reported^26,29,73,74^. Additionally, HilD and PhoP positively and independently regulate the expression of the *slrP* gene, in SPI-1-inducing growth conditions, PhoP directly and HilD by an unknown mechanism^37^. The *slrP* gene encodes a virulence effector protein that is translocated into macrophages through both T3SS-1 and T3SS-2^25^. The overlap between the HilD and PhoP regulons extends to several other genes, as revealed by recent transcriptomic analyses^41^ and by our results (unpublished).

We found that GrhD1 is required for the invasion of *S*. Typhimurium to host cells; interestingly, both the absence or overexpression of GrhD1 inhibit the invasion phenotype. Consistently, we show that GrhD1 is involved in the intestinal inflammatory response induced by *S*. Typhimurium in streptomycin-pretreated mice. Many other genes regulated by HilD, such as the SPI-1 genes, required for the invasion of host cells, also contribute to the induction of intestinal inflammatory response^8,14,15,75^. Our results indicate that GrhD1 is not secreted when *S*. Typhimurium is grown in SPI-1-inducing growth conditions (Fig. S12); on the other hand, the absence of GrhD1 does not affect the typical SPI-1-mediated protein secretion profile or has a significant effect on motility (Fig. S13), which are all factors involved in the *Salmonella* invasion of host cells. Therefore, the specific role of GrhD1 for invasion remains to be defined, which is a matter of our current investigation.

During the growth of *S*. Typhimurium in minimal media, which favor expression of the SPI-2 and other genes required for the *Salmonella* replication inside host cells, the expression of *grhD1* requires PhoP, but not HilD. In SPI-2-inducing growth conditions, PhoP also positively controls transcription of the *orgBC* operon and the *slrP* gene independently of HilD^37,56^. In addition to PhoP, SlyA and possibly other regulators are also involved in the expression of *grhD1* in SPI-2-inducing growth conditions, as revealed by transcriptomic analyses^41^ and confirmed by our results (Fig. S3A). PhoP and SlyA induce expression of virulence genes when *Salmonella* is within host cells^8^; consistently, the *grhD1* gene is expressed inside macrophages^20,76^. Our results indicate that the *grhD1* gene is not necessary for the replication of *S*. Typhimurium inside HeLa cells and RAW264.7 mouse macrophages. Furthermore, our results show that *grhD1* is not required for the colonization of the spleen of streptomycin-pretreated mice, which suggests that it is not involved in the systemic infection caused by *S*. Typhimurium. PhoP positively regulates the *ecgA*-*SL1344_1874* operon, located close to the *grhD1* gene, in the same genomic island; this operon codes for EcgA, a peptidoglycan D,L-endopeptidase that contributes to systemic infection in mice, but it is not required for the invasion of or replication within HeLa cells of *S*. Typhimurium^63^.

Our results indicate that PhoP and SlyA are required for the expression of the *grhD1* gene in both SPI-1-inducing and SPI-2-inducing growth conditions, which could be explained by the reciprocal positive regulation between PhoP and SlyA that has been demonstrated in *Salmonella*^77–80^.

Our findings further expand the HilD, PhoP and SlyA regulons, provide additional evidence on the overlap between these virulence regulons, and reveal a novel virulence factor of *Salmonella*.

## Methods

### Bacterial strains and growth conditions

Bacterial strains used in this work are listed in Table S1. Bacterial cultures were grown at 37 °C in nutrient-rich lysogeny broth (LB), N-minimal medium (N-MM), phosphate-carbon-nitrogen (PCN) minimal medium or intracellular salts medium (ISM) as described previously^81,82^. When needed, the antibiotics ampicillin (200 µg/ml), streptomycin (100 µg/ml), kanamycin (20 µg/ml) or tetracycline (10 µg/ml) were added to the media. The chloramphenicol acetyltransferase (CAT) assays were performed as we described previously^23,83^.

### Construction of plasmids

Tables S1 and S2 indicate the plasmids and primers used in this study, respectively. To construct the plasmids containing the transcriptional fusions pgrhD1-cat, pgrhD1L-cat, pgrhD1 + 1p-cat and pgrhD1 + 1s-cat, different segments of the upstream region of *grhD1* were amplified by PCR with the combination of primers 1872FW-1/1872RV-2, 1872FW-1/1872Rv-3, 1872FW-1/1872Rv + 1s, and 1872Fw + 1p/1872Rv-3, respectively. The generated PCR products were digested with SalI and HindIII restriction enzymes, purified and cloned into the vector pKK232-8, which carries a *cat* reporter gene lacking the promoter (Amersham Pharmacia LKB Biotechnology), digested with the same restriction enzymes. To construct the ppagK-cat plasmid, the upstream region of *pagK* was amplified by PCR with the primers pagKyM-Fw and pagKyM-Rv. This PCR product was digested with BamHI and HindIII restriction enzymes, purified and cloned into the vector pKK232-8 digested with the same restriction enzymes. To construct the pK3-PhoP plasmid, the *phoP* gene was amplified by PCR using the primers PhoP-RV11 and PhoPFW22. This PCR product was digested with BamHI and HindIII restriction enzymes, purified and ligated into the pMPM-K3 vector^84^ digested with the same restriction enzymes. The pK3-PhoP plasmid expresses PhoP from the vector *lac* promoter. To construct the pBAD-H-NS-FH plasmid, the *hns* gene was amplified by PCR using the primers HNS-NcoI and Flag-His, and chromosomal DNA from the EPEC E2348/69 *hns::3xFLAG-kan* strain (V.H. Bustamante, unpublished) as template. This PCR product was digested with NcoI and HindIII restriction enzymes, purified and cloned into the vector pBADMycHisA digested with the same restriction enzymes. The pBAD-H-NS-FH plasmid expresses H-NS fused to 3XFLAG and 6XHis (H-NS-FH) from an arabinose-inducible promoter. To construct the pT6-H-NS-G113D plasmid, the G113D *hns* mutant allele was amplified by PCR using the primers hns-Nco and hns-22R and chromosomal DNA from the *E. coli* HM52 strain^67^ as template. The resulting PCR product was digested with NcoI and HindIII, purified and ligated into the pMPM-T6Ω vector^84^ digested with the same restriction enzymes. The pT6-HNS-G113D plasmid expresses H-NS^G113D^ under an arabinose-inducible promoter. To construct the pK3-GrhD1 and pK3-GrhD1-FLAG plasmids, the *grhD1* gene was amplified by PCR using the primers 1872Fw-K3 and 1872Rv-K3, and chromosomal DNA from the WT and DTM106 (*grhD1::3XFLAG*) *S*. Typhimurium strains, respectively, as template. The PCR products were digested with KpnI and SacI restriction enzymes, purified and ligated into the pMPM-K3 vector digested with the same restriction enzymes. Plasmids pK3-GrhD1 and pK3-GrhD1-FLAG express GrhD1 and GrhD1-FLAG, respectively, from the vector *lac* promoter. To construct the p2795-GrhD1-FLAG plasmid, the *grhD1* gene was amplified by PCR using the primers 1872-SalIFw and 1872Rv-3, and chromosomal DNA from the *S*. Typhimurium DTM106 strain (*grhD1::3XFLAG*) as template. The PCR products were digested with BamHI and SalI restriction enzymes, purified and ligated into the p2795 vector^85^ digested with the same restriction enzymes.

### Chloramphenicol acetyltransferase (CAT) assays

The CAT specific activity was determined as described previously^86^.

### Construction of deletion mutant strains and strains expressing FLAG-tagged proteins

The *grhD1* and *phoP* genes were replaced with a selectable kanamycin resistance cassette in the *S*. Typhimurium SL1344 strain by the λRed recombinase system, as reported previously^87^, thus generating the DTM101 (∆*grhD1::kan*) and DTM103 (∆*phoP::kan*) strains. The chromosomal *grhD1* and *avrA* genes were FLAG-tagged in the *S*. Typhimurium SL1344 strain, using a previously reported method based on the λRed recombinase system^88^, generating the DTM107 (*grhD1::3XFLAG-kan*) and DTM113 (*avrA::3XFLAG-kan*) strains. P22 transduction was used to transfer the ∆*hilD::kan* allele from JPTM5 into DTM104, generating the DTM105 strain, and to transfer the *grhD1::3XFLAG-kan* allele from DTM107 into JPTM25, DTM104, SV4198 and SV4235, generating the DTM109, DTM111, MD3883 and MD3870 strains, respectively. The kanamycin resistance cassette was excised from the DTM101, DTM103, DTM105, DTM107, DTM109 and DTM111 strains, by using helper plasmid pCP20, expressing the FLP recombinase, as described previously^87^, generating the DTM102, DTM104, DTM106, DTM108, DTM110 and DTM112 strains, respectively. The complemented DTM114 strain (∆*grhD1* + *grhD1::3XFLAG-kan*) was generated by inserting in *cis* the *grhD1::3XFLAG-kan* into the chromosome of the DTM102 strain (∆*grhD1*), using a previously reported method based on the λRed recombinase system^85^ and the p2795-GrhD1-FLAG plasmid. All modified strains were verified by PCR amplification and sequencing.

### Western blotting

Whole-cell extracts were prepared from samples of bacterial cultures and analyzed by Western blot as described previously^82^. Antibodies anti-FLAG M2 (Sigma) or anti-GroEL (StressGen) were used at 1:2 000 and 1:100 000 dilutions, respectively. The secondary antibodies Horseradish peroxidase-conjugated anti-mouse or anti-rabbit (Pierce) were used at a dilution of 1:10 000. Reaction bands on membranes were developed with the Western Lightning Chemiluminescence Reagent Plus (Perkin-Elmer) and the exposition to Kodak X-Omat films.

### Protein secretion analysis

Protein secretion assays were performed as described previously^83^.

### Expression and purification of MBP-HilD

The maltose binding protein (MBP)-HilD was expressed and purified from *E. coli* BL21/DE3 containing the pMAL-HilD1 plasmid, using an amylose affinity column, as described previously^23^.

### Expression and purification of PhoP-H6

The His-tagged fusion protein PhoP-H6 was expressed in *E. coli* BL21/DE3 carrying the pPB1020 plasmid and purified by using a Ni^2+^-NTA-agarose affinity column, as described previously^89^.

### Expression and purification of H-NS-FH

The His-tagged fusion protein H-NS-FH was expressed in *E. coli* BL21/DE3 containing the pBAD-H-NS-FH plasmid and purified by using a Ni^2+^-NTA-agarose affinity column, as described previously^23^.

### Electrophoretic mobility shift assays (EMSAs)

For EMSAs with MBP-HilD or H-NS-FH, the upstream regions of *hilA*, *sigD*, *grhD1* and *ssrAB* were amplified by PCR using the combination of primers hilA1FBamHI/hilA2RHindIII, SigDBHIF/SigDH3R, 1872Fw-1/1872Rv-2 (or 1872Fw-1/1872Rv-3) and SsaBFBglII/SsrBRS6E, respectively. The generated PCR products were purified with the QIAquick PCR purification kit (Qiagen). Binding reactions were performed by mixing each PCR product (≈100 ng) with increasing concentrations of purified MBP-HilD or H-NS-FH in binding buffer containing 10 mM Tris-HCl (pH 8), 50 mM KCl, 1 mM DTT, 0.5 mM EDTA, 5% glycerol and 10 µg/ml bovine serum albumin (BSA), in a total volume of 20 µl. These reactions were incubated at room temperature for 20 min and then analyzed by electrophoresis on 6% nondenaturing acrylamide gels ran with 0.5X Tris-borate-EDTA buffer, at room temperature. The DNA fragments were visualized by staining with ethidium bromide, in an Alpha-Imager UV transilluminator (Alpha Innotech Corp.).

For EMSAs with PhoP-H6, the primers that anneal to the coding strand of the promoters analyzed were labeled with T4 polynucleotide kinase and [γ-^32^P] ATP. The promoter regions of *grhD1*, *orgB*, *ges*, and *nucA* were amplified by PCR using primer pairs stm1939 Fwd/stm1939 rv, orgB PE 3/PROM 2869, ges1/ges2, and nucA FW/nuclease RV, respectively. Approximately 6 fmol of labeled promoter region DNA in a 20-µl volume was incubated at room temperature for 30 min with the indicated amounts of purified PhoP-H6 protein, which was previously phosphorylated by incubation for 3 h at 25 °C with 25 mM acetyl phosphate as reported^90^. The binding buffer used for protein-DNA incubations contained 20 mM Tris-HCl (pH 7.4), 50 mM KCl, 5 mM MgCl_2_, 10% glycerol, and 25 µg/ml BSA. Samples were separated in an 8% nondenaturing Tris-borate-EDTA–polyacrylamide gel at room temperature. After electrophoresis, the gel was dried and analyzed in a Typhoon FLA 7000 IP laser scanner.

### Motility assays

*Salmonella* strains were grown overnight at 37 °C with appropriate antibiotics. Then, the strains were sub-cultured 1:100 in fresh LB and grown at 37 °C with shaking until an OD_600_ ∼ 1. At this point, 1 µl of each culture was spotted onto LB 0.3% agar plates and allowed to dry for 3 min at room temperature. Plates were incubated for 7 h at 37 °C and the diameter of the motility haloes was measured.

### Cell infection assays

Invasion of HeLa cells or RAW264.7 macrophages was tested by gentamicin assays as previously described^69,82^. Monolayers of HeLa cells or RAW264.7 macrophages were infected at a multiplicity of infection (MOI) of 40:1 and 10:1 (bacteria to eukaryotic cells), respectively. In some experiments, monolayers were centrifuged at 1000 g for 10 min immediately after addition of the bacteria and then incubated for 10 min at 37 °C. To test the intracellular replication/survival, the monolayers of HeLa cells or RAW264.7 macrophages were further incubated with DMEM containing 10 µg/ml gentamicin up to the indicated times. After removing the DMEM, the cells were lysed at 1, 4, 8, and 16 h post-infection in 1 ml or 200 µl of 0.2% (w/v) sodium deoxycholate in 1X PBS for HeLa cells and RAW264.7 macrophages, respectively. Serial dilutions of the cell lysates were plated onto LB agar containing streptomycin at 100 µg/ml. To evaluate invasion, the CFUs were counted at 1 h post-infection; to test intracellular replication/survival, the CFUs were enumerated at 4, 8, or 16 h post-infection. Fold-replication represents the CFUs recovered at 4, 8, or 16 h relative to the CFUs at 1 h post-infection.

The fibroblast cell line NRK-49F (ATCC CRL-1570) of rat origin, were propagated in DMEM containing 10% (v/v) fetal bovine serum, as described previously^91^. For the invasion assay, bacteria were grown at 37 °C in static non-aerated cultures obtained upon inoculation of 2 ml of LB with a bacterial colony and subsequent overnight incubation (final OD_600_ ~ 1.0). Infection was carried out for either 10 or 20 min using a MOI of 10:1, as previously described^91^. After extensive washing, fibroblasts were incubated in fresh tissue culture medium containing 100 µg/ml gentamicin up to 2 h post-infection. At that time, fibroblasts were lysed in 1X PBS pH 7.4, 1% (v/v) Triton X-100. Number of viable intracellular bacteria was determined by plating.

### Mouse infection experiments

Animal manipulation in this work was carried out according to the standard and operating protocols approved by the Internal Committee for Animal Care and Use from CICUAL-UNAM, and by the Official Mexican Norm NOM-062Z00-1999. Pathogen-free BALB/c mice (6- to 7-week-old) were obtained from the Experimental Medicine Research Unit, School of Medicine, UNAM, Mexico. Groups of four animals were maintained in different ventilated cages. Water and food were withdrawn 4 h before treatment of mice with 50 mg of streptomycin by orogastric administration; then, animals were supplied with water and food ad libitum. For infection, overnight cultures of the *Salmonella* strains were diluted 1:100 in 5 ml of fresh LB and incubated at 37 °C with shaking for 3 h. After 24 h of the streptomycin treatment, water and food were withdrawn again for 4 h before the infection of mice with 50 µl of a bacterial suspension containing 1 × 10^8^ CFUs/ml in 1X PBS, or the administration of 50 µl of sterile 1X PBS (control). Thereafter, drinking water and food were offered to the animals ad libitum. At 48 h post-infection, the animals were euthanatized with an overdose of the anesthetics

Ketamine plus Xylazine administrated intraperitoneally, in a workstation hood (Thermo-Scientific). The spleen, cecum and terminal ileum were aseptically removed, weighed and homogenized in 1 ml of sterile and cold 1X PBS. To evaluate infiltration of neutrophils, samples of the cecum contents were analyzed by microscopy to determine the number of neutrophils and total eukaryotic cells from 15 different fields, using the Diff-Quick staining method and an inverted microscope Nikon TE300 (objective 60X). To analyze bacterial colonization, 2% Triton X-100 was added to the organ homogenizates and CFUs were determined by plating serial dilutions of the obtained cell lysates onto LB agar containing streptomycin at 100 µg/ml.

### Statistical analysis

Data were analyzed with the GraphPad Prism 5.0 software (GraphPad Inc., San Diego, CA) using two-tailed Student’s *t*-test. *P* values of <0.05 were considered significant.

## Electronic supplementary material

Supplementary Information
