# Publisher Correction: HilD and PhoP independently regulate the expression of *grh*D1, a novel gene required for *Salmonella* Typhimurium invasion of host cells

**DOI:** 10.1038/s41598-018-25327-6

**Published:** 2018-05-11

**Authors:** María M. Banda, Carolina López, Rubiceli Manzo, Gadea Rico-Pérez, Pablo García, Roberto Rosales-Reyes, Miguel A. De la Cruz, Fernando C. Soncini, Francisco García-del Portillo, Víctor H. Bustamante

**Affiliations:** 1 Departamento de Microbiología Molecular, Instituto de Biotecnología, Universidad Nacional Autónoma de México, Cuernavaca, Morelos 62210 Mexico; 2 Instituto de Biología Molecular y Celular de Rosario, Universidad Nacional de Rosario, Rosario, Argentina; 3 Laboratorio de Patógenos Bacterianos Intracelulares, Centro Nacional de Biotecnología-Consejo Superior de Investigaciones Científicas (CNB-CSIC), Madrid, Spain; 4 Unidad de Investigación en Medicina Experimental, Facultad de Medicina, Universidad Nacional Autónoma de México, Ciudad de México, Mexico; 5 Unidad de Investigación Médica en Enfermedades Infecciosas y Parasitarias, Hospital de Pediatría, Centro Médico Nacional Siglo XXI, Instituto Mexicano del Seguro Social, Ciudad de México, Mexico

Correction to: *Scientific Reports* 10.1038/s41598-018-23068-0, published online 19 March 2018

In the original version of this Article, the author Francisco García-del Portillo was incorrectly indexed. This error has now been corrected.

