# Supplementary material for: HilD and PhoP independently regulate the expression of grhD1, a novel gene required for Salmonella Typhimurium invasion of host cells
Source: Sci Rep. 2018 Mar 19;8:4841. doi: 10.1038/s41598-018-23068-0 (PMC5859253; doi:10.1038/s41598-018-23068-0)
Supplement: Supplementary file 1 — Supplementary Information [file 41598_2018_23068_MOESM1_ESM.pdf]

Supplementary Information

HilD and PhoP independently regulate the expression of *grhD1*, a novel gene required for *Salmonella* Typhimurium invasion of host cells

María M. Banda, Carolina López, Rubiceli Manzo, Gadea Rico-Pérez, Pablo García, Roberto Rosales-Reyes, Miguel A. De la Cruz, Fernando C. Soncini, Francisco García-del Portillo & Víctor H. Bustamante

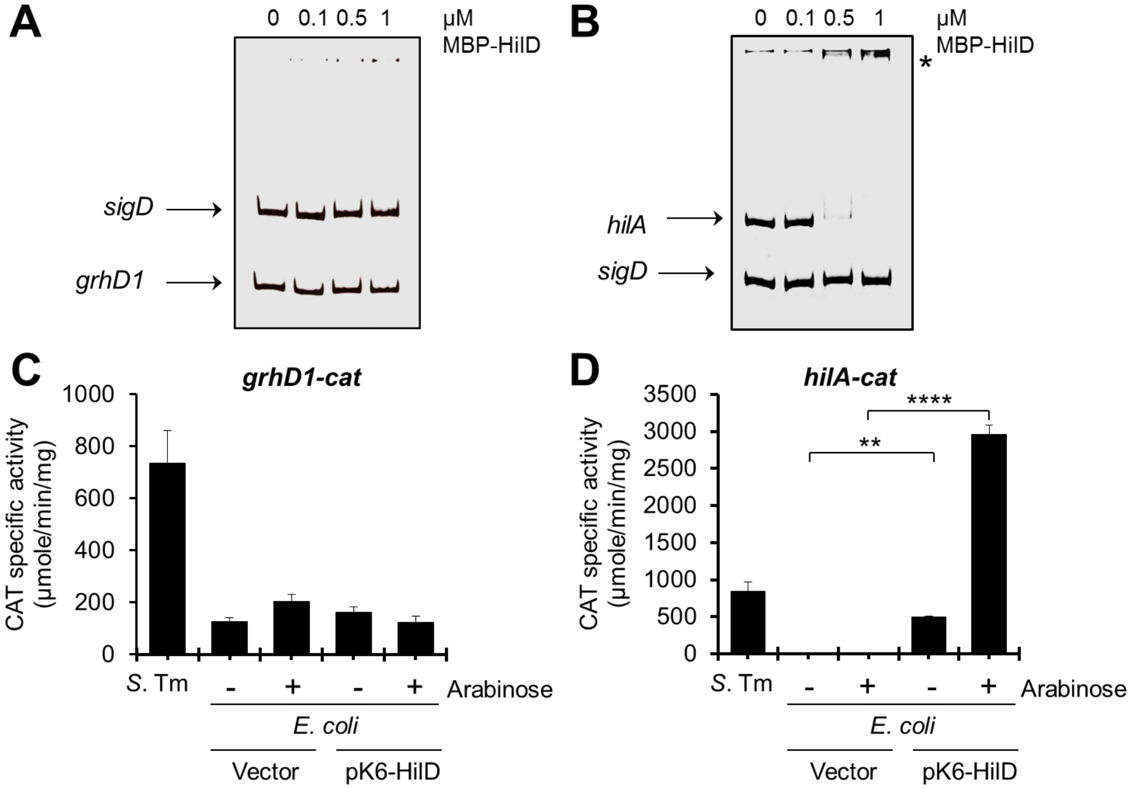

Figure S1. HilD indirectly regulates the expression of *grhD1*. EMSAs with purified MBP-HilD (0, 0.1, 0.5 and 1 µM) and DNA fragments containing the regulatory region of *grhD1* (A) and *hilA* (B). These DNA fragments correspond to those contained in the *grhD1-cat* and *hilA-cat* transcriptional fusions. A DNA fragment containing the regulatory region of *sigD* was used as a negative internal control. The DNA-protein complexes, which are indicated by an asterisk, were resolved in a nondenaturing 6% polyacrylamide gel and stained with ethidium bromide. Expression of the *grhD1-cat* (C) and *hilA-cat* (D) transcriptional fusions, contained in the pgrhD1-cat and philA-cat plasmids, respectively, was tested in the WT *E. coli* MC4100 strain containing the pK6-HilD plasmid expressing HilD from an arabinose-inducible promoter, or the pMPM-K6Ω vector. CAT specific activity was determined from samples collected of bacterial cultures grown in LB at 37°C. 0.001% L-arabinose was added (+) or not (-) to the medium for inducing the expression of HilD from pK6-HilD. The data are the average of three independent experiments performed in duplicate. Bars represent the standard deviations. Statistically different values are indicated (**, $p<0.01$; ****, $p<0.0001$).

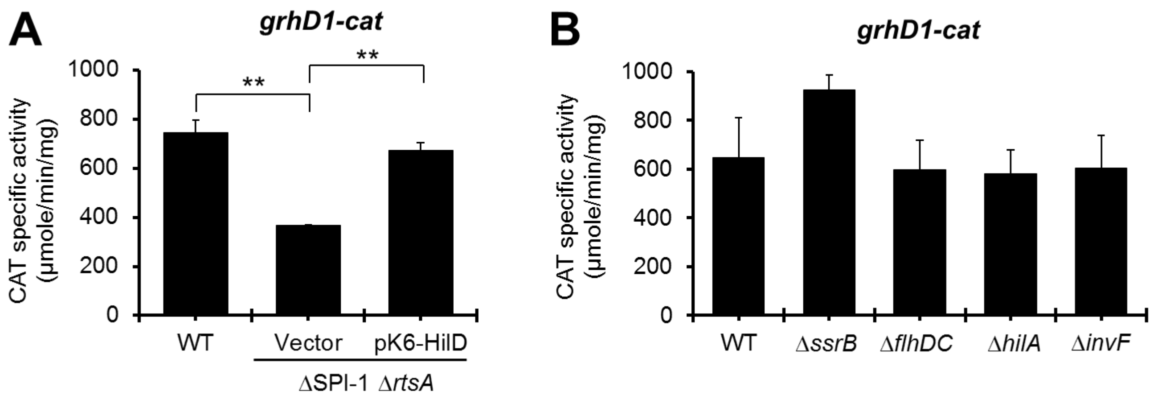

Figure S2. The HilD-mediated expression of *grhD1* does not require any other regulator encoded in SPI-1, neither RtsA, SsrB or FlhDC. Expression of the *grhD1-cat* transcriptional fusion contained in the pgrhD1-cat plasmid, was tested in the WT *S*. Typhimurium strain and its isogenic ΔSPI-1 Δ*rtsA* mutant containing the pK6-HilD plasmid expressing HilD, or the pMPM-K6Ω vector (A); as well as in the WT *S*. Typhimurium strain and its isogenic Δ*ssrB*, Δ*flhDC*, Δ*hilA* and Δ*invF* mutants (B). CAT specific activity was determined from samples collected of bacterial cultures grown in LB at 37°C. The data are the average of three independent experiments performed in duplicate. Bars represent the standard deviations. Statistically different values are indicated (**, $p<0.01$).

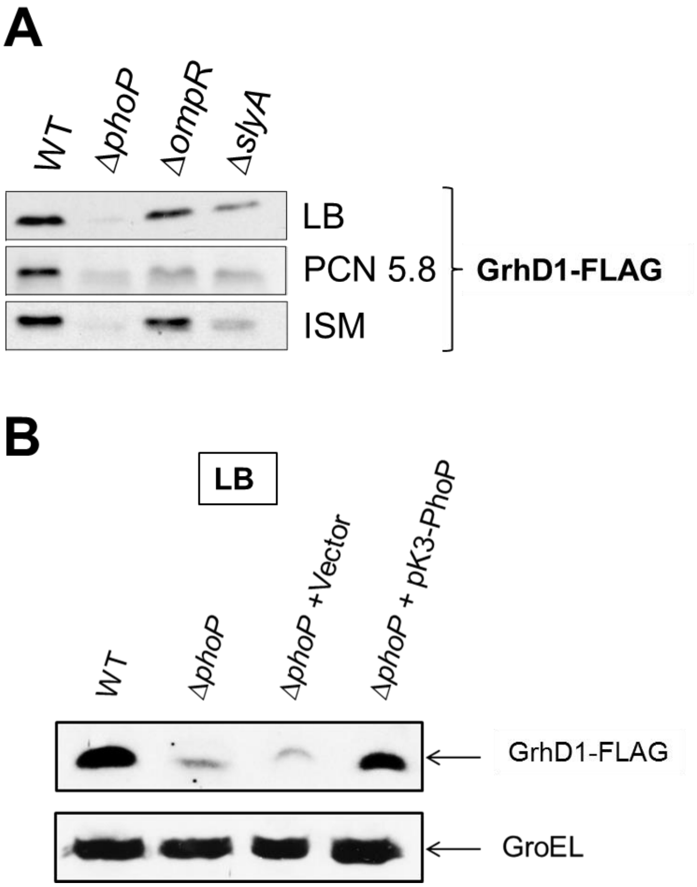

Figure S3. PhoP and SlyA positively regulate the expression of GrhD1 in different growth conditions. Expression of GrhD1-FLAG was tested in the WT *S*. Typhimurium strain and isogenic Δ*phoP*, Δ*ompR* and Δ*slyA* mutants grown in LB, acidic PCN medium or ISM, at 37°C (A); as well as in the WT *S*. Typhimurium strain and its isogenic Δ*phoP* mutant containing or not the pK3-PhoP plasmid constitutively expressing PhoP, or the pMPM-K3 vector, grown in LB. Whole-cell lysates were prepared from samples of the bacterial cultures and analyzed by Western blotting using monoclonal anti-FLAG antibodies. As a loading control, the expression of GroEL was also detected using polyclonal anti-GroEL antibodies.

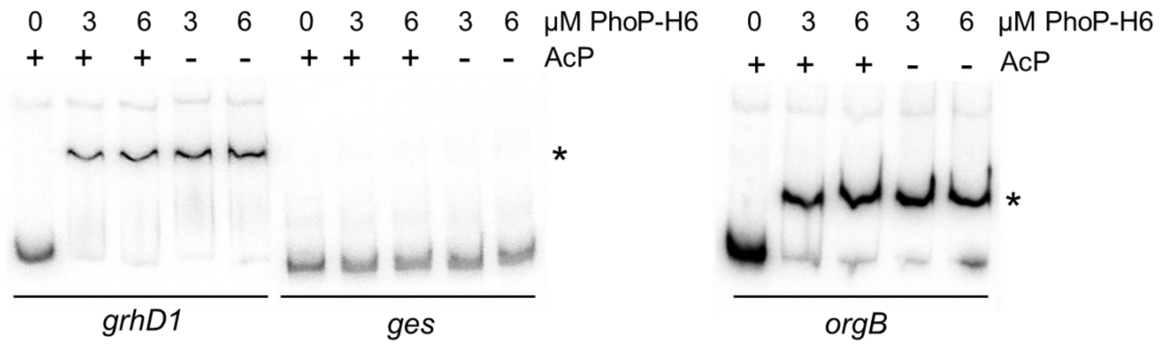

Figure S4. PhoP binds to the *grhD1* regulatory region. EMSAs with purified PhoP and DNA fragments containing the regulatory regions of the *grhD1*, *ges* or *orgB* genes. ^{32}P-5'-end-labelled DNA fragments of the respective gene were incubated with increasing concentrations of PhoP-H6 (0, 3 and 6 µM) in the presence (+) or absence (-) of acetyl-phosphate, AcP. The *ges* and *orgB* genes were used as negative and positive controls, respectively. The DNA-protein complexes, which are indicated by an asterisk, were resolved in a nondenaturing 8% Tris-borate-EDTA-polyacrylamide gel. After electrophoresis, the gel was dried and analyzed in a Typhoon FLA 7000 IP laser scanner.

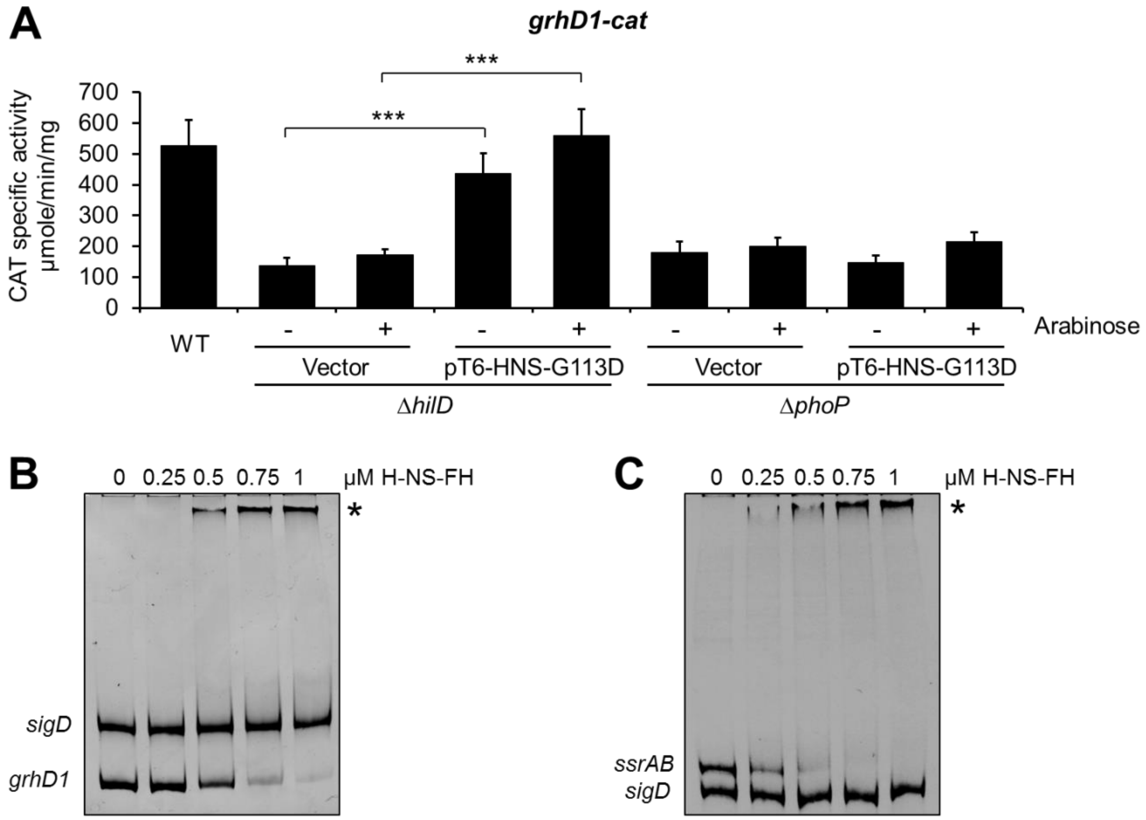

Figure S5. H-NS directly represses the expression of *grhD1*. Expression of the *grhD1-cat* transcriptional fusion (A) contained in the pgrhD1-cat plasmid was analyzed in the WT *S*. Typhimurium strain and its isogenic Δ*hilD* and Δ*phoP* mutants containing the pT6-HNS-G113D plasmid expressing the dominant negative H-NS^{G113D} mutant, or containing the pMPM-T6Ω vector. CAT specific activity was determined from samples collected of bacterial cultures grown in LB at 37°C. 0.1% L-arabinose was added (+) or not (-) to the medium for inducing the expression of H-NS^{G113D} from pT6-HNS-G113D. The data are the average of three independent experiments performed in duplicate. Bars represent the standard deviations. Statistically different values are indicated (***, $p<0.001$). EMSAs with purified H-NS-FH (0, 0.25, 0.5, 0.75 and 1 µM) and DNA fragments containing the regulatory region of *grhD1* (B) or *ssrAB* (C). A DNA fragment containing the regulatory region of *sigD* was used as a negative internal control. The DNA-protein complexes, which are indicated by an asterisk, were resolved in a nondenaturing 6% polyacrylamide gel and stained with ethidium bromide.

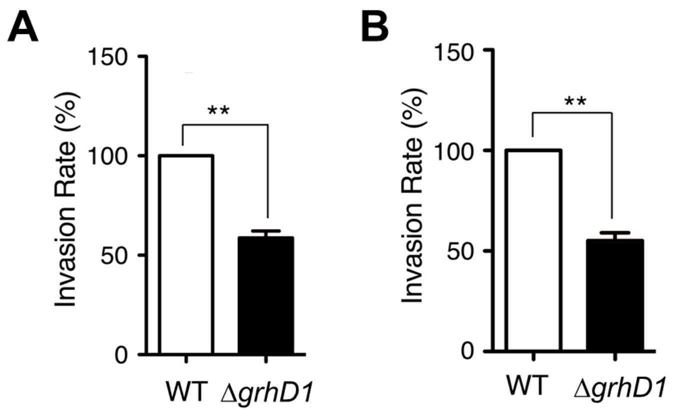

Figure S6. GrhD1 is required for invasion of *S*. Typhimurium into fibroblasts. NRK-F9 fibroblasts were infected with the WT *S*. Typhimurium strain or its isogenic Δ*grhD1* mutant for 10 min (A) or 20 min (B). Invasion was measured by enumerating the intracellular CFUs at 1 h post-infection, using a gentamicin protection assay, and represented as the percent with respect to the WT strain. Results are the mean of three separate experiments each in triplicate. Bars represent the standard deviations. Statistically different values are indicated (**, p<0.01).

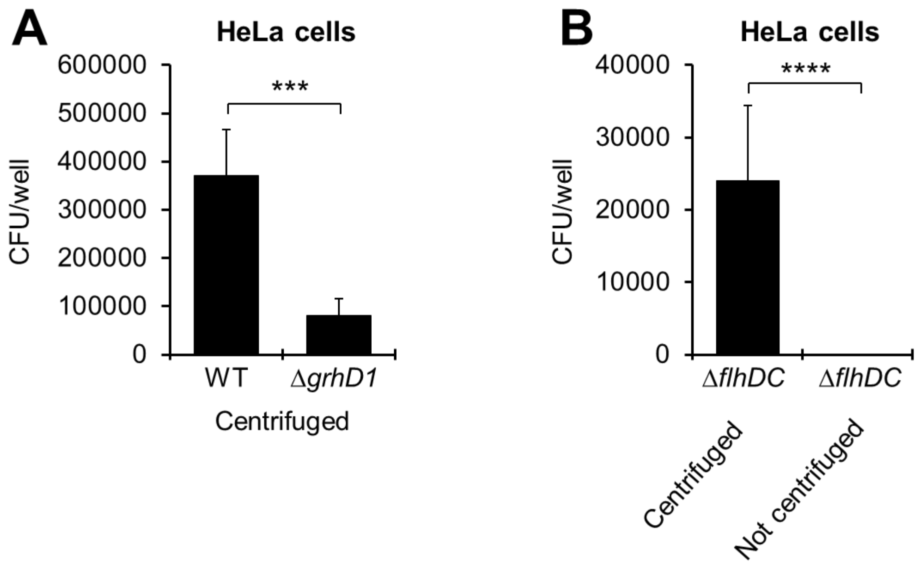

Figure S7. Motility is not involved in the invasion defect of the Δ*grhD1* mutant in HeLa cells. HeLa cells were infected with the WT *S*. Typhimurium strain or its isogenic Δ*grhD1* (A) and Δ*flhDC* (B) mutants, either with or without centrifugation. Invasion was measured by enumerating the intracellular CFUs at 1 h post-infection, using a gentamicin protection assay. Results are the mean of three separate experiments each in triplicate. Bars represent the standard deviations. Statistically different values are indicated (***, $p<0.001$; ****, $p<0.0001$).

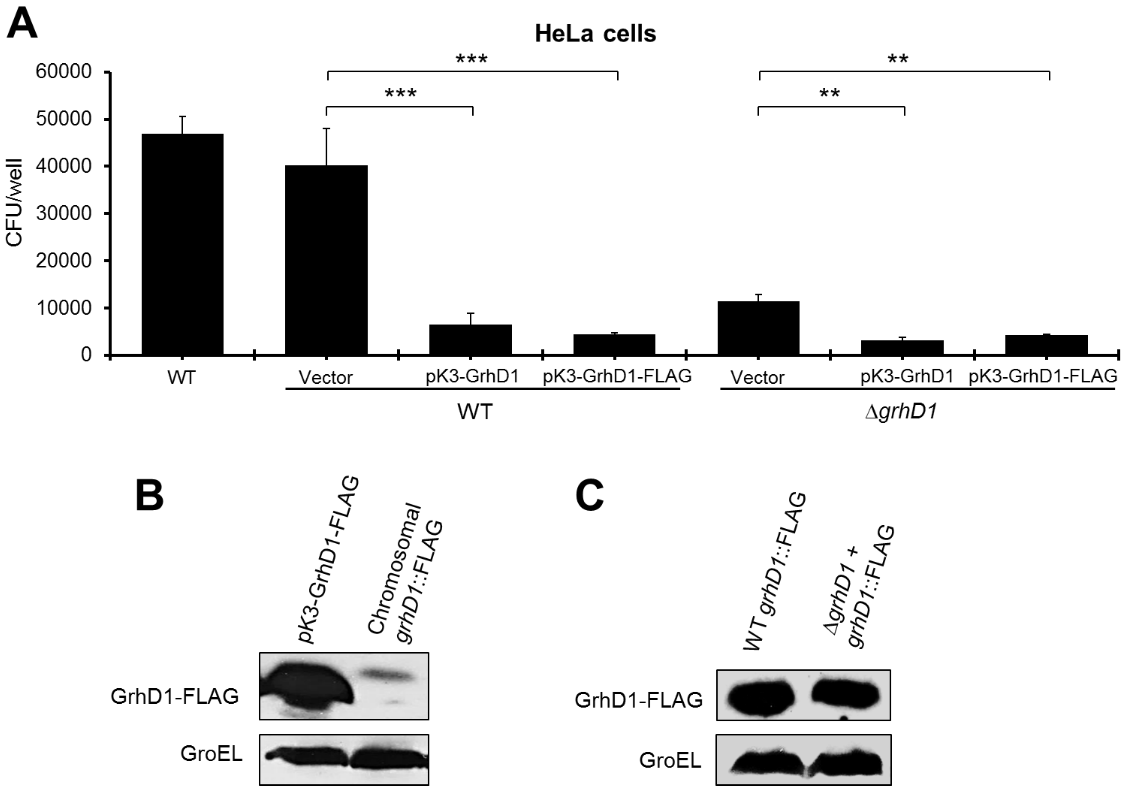

Figure S8. Overexpression of GrhD1 decreases invasion of *S*. Typhimurium into HeLa cells. (A) HeLa cells were infected with the WT *S*. Typhimurium strain or its isogenic Δ*grhD1* mutant strain, containing or not the pK3-GrhD1 or pK3-GrhD1-FLAG plasmids, which express GrhD1 and GrhD1-FLAG proteins, respectively, from a constitutive *lac* promoter, or containing the pMPM-K3 vector. Invasion was measured by enumerating the intracellular CFUs at 1 h post-infection, using a gentamicin protection assay. Results are the mean of three separate experiments each in triplicate. Bars represent the standard deviations. Statistically different values are indicated (**, p<0.01; ***, p<0.001). Expression of the FLAG-tagged *grhD1* gene carried by pK3-GrhD1-FLAG and that located in the chromosome of the WT *S*. Typhimurium (B), or expression of the FLAG-tagged *grhD1* gene located in the chromosome of the WT *S*. Typhimurium and that inserted in the chromosome of the Δ*grhD1* strain (C), was analyzed by Western blotting using anti-FLAG monoclonal antibodies. Whole-cell extracts used in these assays were prepared from samples of bacterial cultures grown in LB at 37°C. As a loading control, the expression of GroEL was also tested using polyclonal anti-GroEL antibodies.

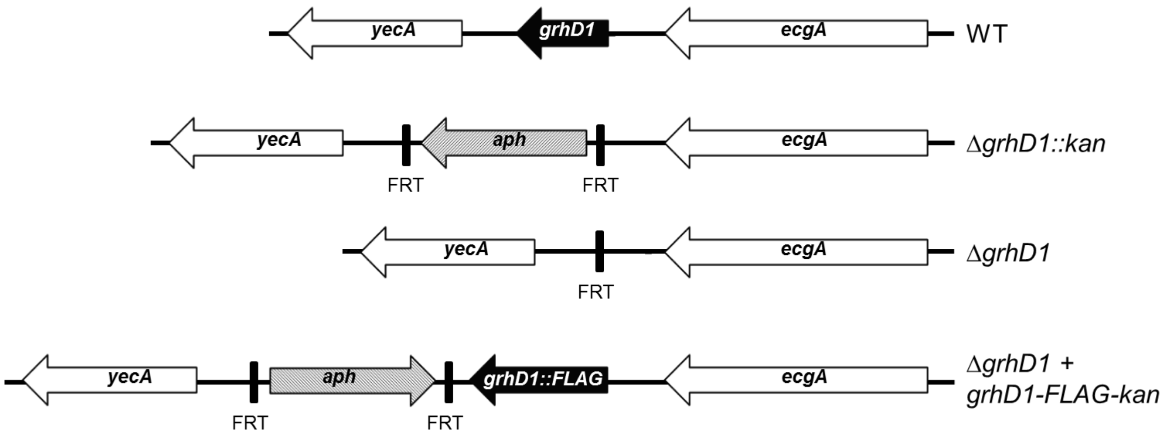

Figure S9. Generation of the Δ*grhD1* mutant strains and the complemented Δ*grhD1* + *grhD1-FLAG-kan* strain. The Δ*grhD1::kan* mutant was obtained by replacing *grhD1* in the WT *S*. Typhimurium strain with a kanamycin resistance gene, using the λRed recombinase; then, the kanamycin gene was excised from the Δ*grhD1::kan* mutant, using the FLP recombinase, to obtain the Δ*grhD1* mutant. The complemented Δ*grhD1* + *grhD1-FLAG-kan* strain was obtained by inserting the *grhD1-FLAG* gene, together with the kanamycin resistance gene in the opposite direction, into the chromosome of the Δ*grhD1* mutant*,* using the λRed recombinase.

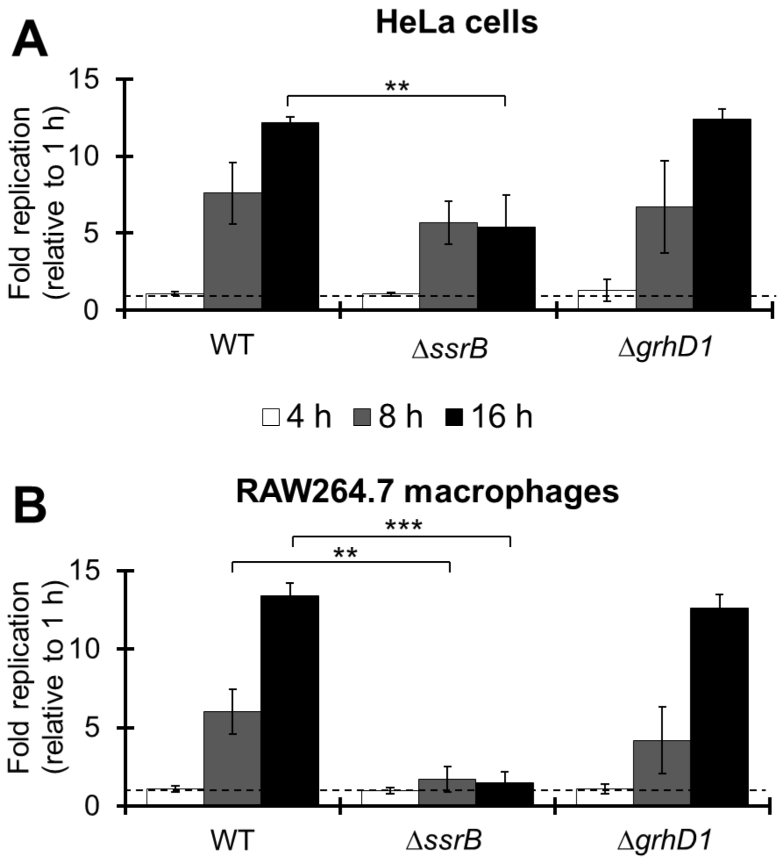

Figure S10. GrhD1 is not required for replication/survival of *S*. Typhimurium inside HeLa cells and macrophages. HeLa cells (A) and murine RAW 264.7 macrophages (B) were infected with the WT *S*. Typhimurium strain or its isogenic Δ*grhD1* and Δ*ssrB* mutants. Intracellular CFUs of each strain were determined from lysates of infected cells at 1, 4, 8 and 16 h post-infection. Fold-replication represents the CFUs recovered at the indicated time points divided by the CFUs at 1 h post-infection, for each strain. The dashed line indicates a value of 1. Results are the mean of three separate experiments each in triplicate. Bars represent the standard deviations. Statistically different values are indicated (**, $p<0.01$; ***, $p<0.001$).

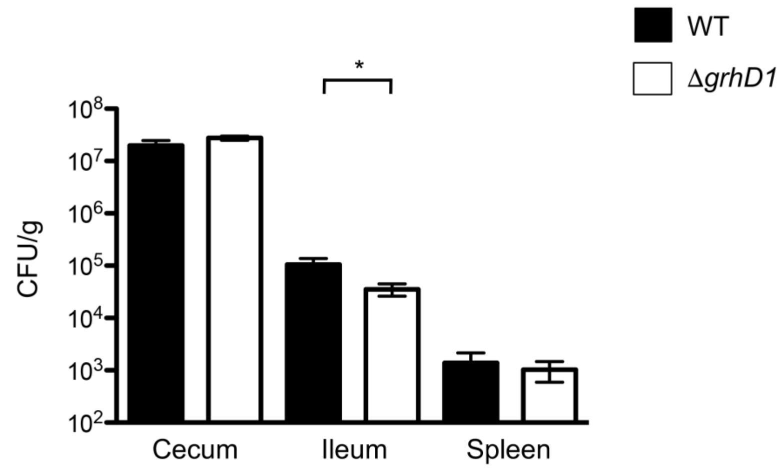

Figure S11. Contribution of GrhD1 on the colonization of the intestine and the spleen of mice by *S.* Typhimurium. Mice pretreated with streptomycin were infected with the WT *S.* Typhimurium strain or the Δ*grhD1* mutant. The mice were sacrificed two days post-infection and the CFUs per gram of cecum, ileum and spleen, from each mouse, were determined. Results shown represent the means and standard deviations from four separate animals. Statistically different values are indicated (*, $p<0.05$).

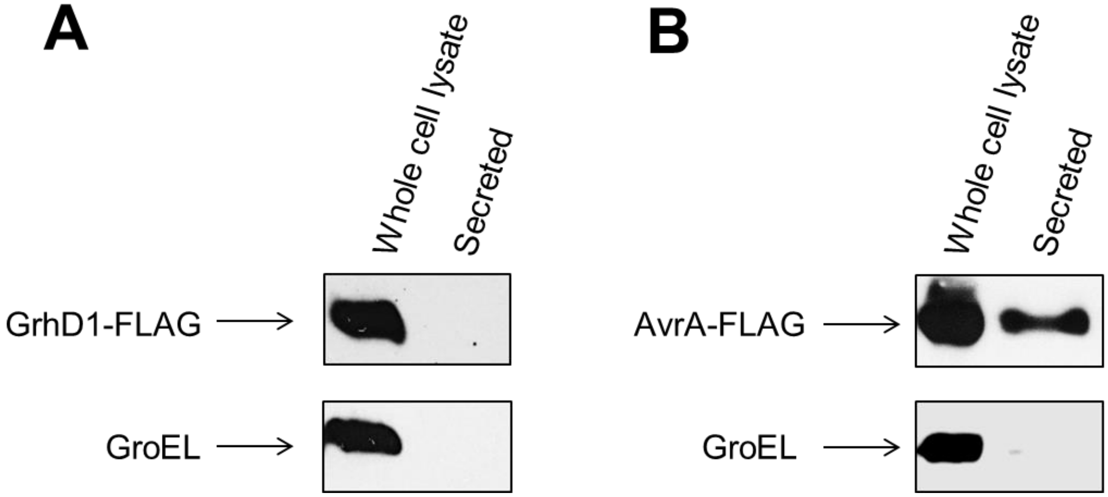

Figure S12. GrhD1 is not secreted. Detection of GrhD1-FLAG from whole-cell extracts and secreted proteins of the WT *S*. Typhimurium strain containing the pK3-GrhD1-FLAG plasmid, which expresses GrhD1-FLAG from a constitutive *lac* promoter (A). Detection of AvrA-FLAG, an effector protein secreted by the T3SS-1, from whole-cell extracts and secreted proteins of the WT *S*. Typhimurium strain carrying a chromosomal FLAG-tagged *avrA* gene (B). Samples were prepared from bacterial cultures grown in LB at 37°C and analyzed by Western blot using anti-FLAG monoclonal antibodies. As a control, detection of the cytoplasmic protein GroEL was also tested using polyclonal anti-GroEL antibodies.

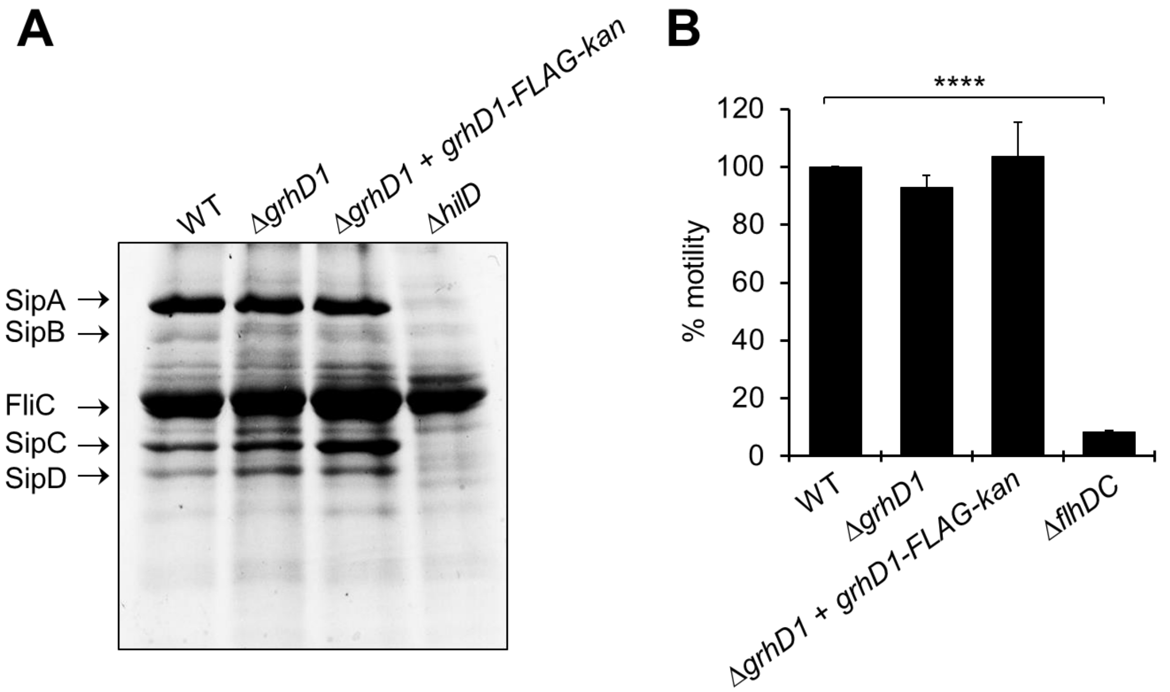

Figure S13. GrhD1 is not required for the activity of the T3SS-1 nor for motility. (A) Protein secretion profiles of the WT *S*. Typhimurium strain and its isogenic Δ*grhD1* and Δ*grhD1* + *grhD1-FLAG-kan* strains. SipA, SipB, SipC and SipD are proteins encoded in SPI-1 and secreted by the T3SS-1. As a control, the Δ*hilD* mutant, which does not express the SipA-D proteins, was also tested in these assays. FliC is a flagellar protein whose secretion is not SPI-1 dependent. (B) Motility of the WT *S*. Typhimurium strain and its isogenic Δ*grhD1* and Δ*grhD1* + *grhD1-FLAG-kan* strains. The Δ*flhDC* mutant lacking FlhDC, the central positive regulator for the flagellar genes, was also assessed as a positive control. The strains were inoculated onto LB 0.3% agar plates and incubated at 37°C for 7 h. The distance (mm) migrated by each mutant strain was compared with that of the WT strain, which is represented as the percent with respect to the WT strain. The data are the average from five independent experiments. Bars represent the standard deviations. Statistically different values are indicated (****, p<0.0001).

Strain or plasmid	Genotype or description	Source or reference
Strain		
S. Typhimurium		
SL1344	Wild type; *xyl*, *hisG*, *rpsL*; Sm^R	1
DTM97	Δ*ssrB::kan*	2
VV341	Δ*hilA::kan-339*	3
JPTM4	Δ*invF::kan*	4
JPTM5	Δ*hilD::kan*	4
JPTM25	Δ*hilD*	5
DTM90	Δ*flhDC::kan*	6
DTM94	Δ*rtsA* ΔSPI-1	2
SV4198	Δ*slyA*	7
MD3883	*grhD1::3XFLAG-kan* Δ*slyA*	This study
SV4235	Δ*ompR*	7
MD3870	*grhD1::3XFLAG-kan* Δ*ompR*	This study
DTM101	Δ*grhD1::kan*	This study
DTM102	Δ*grhD1*	This study
DTM103	Δ*phoP::kan*	This study
DTM104	Δ*phoP*	This study
DTM105	Δ*phoP* Δ*hilD::kan*	This study
DTM106	Δ*phoP* Δ*hilD*	This study
DTM107	*grhD1::3XFLAG-kan*	This study
DTM108	*grhD1::3XFLAG*	This study
DTM109	*grhD1::3XFLAG-kan* Δ*hilD*	This study
DTM110	*grhD1::3XFLAG* Δ*hilD*	This study
DTM111	*grhD1::3XFLAG-kan* Δ*phoP*	This study
DTM112	*grhD1::3XFLAG* Δ*phoP*	This study
DTM113	*avrA::3XFLAG-kan*	This study
DTM114	Δ*grhD1* + *grhD1::3XFLAG-kan*	This study
E. coli		
MC4100	F^-(*araD139*) Δ(*argF-lac*)*169*λ^- $e14^-$*flhD5301* Δ(*fruK-yeiR*)725(*fruA25*) *relA1 rpsL150*(Str^r) *rbsR22* Δ(*fimB-fimE*)*632*(::IS*1*) *deoC1*	8
DH10β	Laboratory strain	Invitrogen
BL21/DE3	Strain for expression of recombinant proteins	Invitrogen
Plasmids		
pKK232-8	pBR322 derivative containing a promotorless chloramphenicol acetyltransferase (*cat*) gene, Ap^R	9
pgrhD1-cat	pKK232-8 derivative containing a	

	grhD1-cat transcriptional fusion from nucleotides -61 to +270	This study
pgrhD1L-cat	pKK232-8 derivative containing a *grhD1-cat* transcriptional fusion from nucleotides -250 to +270	This study
pgrhD1+1p-cat	pKK232-8 derivative containing a *grhD1-cat* transcriptional fusion from nucleotides -250 to +27	This study
pgrhD1+1s-cat	pKK232-8 derivative containing a *grhD1-cat* transcriptional fusion from nucleotides +16 to +270	This study
ppagK-cat	pKK232-8 derivative containing a *pagK-cat* transcriptional fusion from nucleotides -880 to +251	This study
philA-cat	pKK232-8 derivative containing a *hilA-cat* transcriptional fusion from nucleotides -410 to +446	[4]
psirA-cat	pKK232-8 derivative containing a *sirA-cat* transcriptional fusion from nucleotides -563 to +98	[5]
pKD46	pINT-ts derivative expressing red recombinase under an arabinose-inducible promoter, Ap^R	[10]
pKD4	pANTsγ derivative template plasmid containing the kanamycin cassette for λRed recombination, Ap^R	[10]
pCP20	Plasmid expressing FLP recombinase from a temperature-inducible promoter, Ap^R	[10]
pSUB11	pGP704 derivative template plasmid for FLAG epitope tagging	[11]
pMAL-HilD1	pMAL-c2X derivative expressing MBP-HilD from a *lac* promoter, Ap^R	[4]
pBADMycHisC	Expression vector for constructing C-terminal MycHis fusions, *ara* promoter, Ap^R	Invitrogen
pBAD-H-NS-FH	pBADMycHisC derivative expressing H-NS-FH from an *ara* promoter, Ap^R	This study
pPB1020	pT7-7 derivative expressing PhoP-H6 under the control of the T7 φ10 promoter, Ap^R	[12]
pMPM-K3	p15A derivative low-copy-number	

	cloning vector, *lac* promoter, Kan^R	[13]
pK3-PhoP	pMPM-K3 derivative expressing PhoP from the *lac* promoter	This study
pK3-SirA	pMPM-K3 derivative expressing SirA from the *lac* promoter	[5]
pK3-GrhD1	pMPM-K3 derivative expressing GrhD1 from the *lac* promoter	This study
pK3-GrhD1-FLAG	pMPM-K3 derivative expressing GrhD1-FLAG from the *lac* promoter	This study
pMPM-K6Ω	p15A derivative cloning vector containing an arabinose-inducible promoter, Kan^R	[13]
pK6-HilD	pMPM-K6Ω derivative expressing HilD under an arabinose-inducible promoter	[6]
pMPM-T6Ω	p15A derivative cloning vector containing an arabinose-inducible promoter, Tc^R	[13]
pT6-HNS-G113D	pMPM-T6Ω derivative expressing H-NS^{G113D} from the arabinose-inducible promoter	This study
p2795	pBluescript SK+ containing *aph* FRT, Ap^R Kan^R	[14]
p2795-GrhD1-FLAG	p2795 containing *grhD1::3XFLAG*	This study

Table S1. Bacterial strains and plasmids used in this study. The coordinates for the *cat* fusions are indicated with respect to the primary transcriptional start site reported for each gene. Ap^R, ampicillin resistance; Sm^R, streptomycin resistance; Kan^R, kanamycin resistance.

Primer	Sequence (5’-3’)	Target gene	RE
**For *cat* transcriptional fusions**			
1872FW-1	CAGGTCGACAAGATCGCGCTCTGACCG	*grhD1*	SalI
1872RV-2	TGAGGATCCTGAGCTGATATCACTGGC	*grhD1*	BamHI
1872Fw+1p	GCCGTCGACATGTCTTCCGCGATAACACAG	*grhD1*	SalI
1872Rv+1s	CTGGGATCCCGCGGAAGACATGATTAAGGC	*grhD1*	BamHI
1872Rv-3	CAGGGATCCAAAACCGTTTACTACGCTGGTC	*grhD1*	BamHI
hilA1FBamHI	ATCGGATCCCTCTGAGAACTATTTGC	*hilA*	BamHI
hilA2RHindIII	GACAAGCTTTTCTGAGCGTAGCAGGG	*hilA*	HindIII
pagKyM-Fw	CGAAAGCTTAGATCTATGATCTTGAGAGTC<br>TGCCG	*pagK*	HindIII
pagKyM-Rv	CGAAAGCTTGGATCCTTGCACAGTCTGCA<br>GTGCTA	*pagK*	BamHI
SirAF-BamHI	GCCGGATCCATCGCCTGCAGCATCAGC	*sirA*	BamHI
SirAR-HindIII	AGCAAGCTTCACCGACAACTTTAATGC	*sirA*	HindIII
**For gene deletions**			
SL1872H1P1	TGTTGGCGGTATCTGGCTGGAGGCTGGG<br>TGGTAAAAAGACGT*TGTAGGCTGGAGCT*<br>*GCTTCG*	*grhD1*	
SL1872H2P2	TATACTCATGCTAACTTATACCACCGCCG<br>CGGACAGATAAAA*CATATGAATATCCTCC*<br>*TTAG*	*grhD1*	
SphoP-H1P1	TAACACAAGGGAGAAGAGATGATGCGCG<br>TACTGGTTGTAGAG*TGTAGGCTGGAGCTG*<br>*CTTCG*	*phoP*	
SphoP-H2P2	TCCGCGTACGGTGGTAATGACATCGTGC<br>GGATACTGGGCCTG*CATATGAATATCCT*<br>*CCTTAG*	*phoP*	
**For gene FLAG tagging**			
SL1872FLAG-F	CTCTATTTGAGAGGGCGGTTTTATCTGT<br>CCGCGGCGGTGGTA*GACTACAAAGACC*<br>*ATGACGG*	*grhD1*	
SL1872FLAG-R	AAGCGAGAGATGGGTTTCTCGCTATATT<br>ATACTCATGCTAAC*CATATGAATATCCTC*<br>*CTTAG*	*grhD1*	
AvrAFLAG-F	CATAAAAAAAGGATAGCTGAATATAAGT<br>CTTTACTTAAACCG*GACTACAAAGACCA*<br>*TGACGG*	*avrA*	
AvrAFLAG-R	GCGCTGGAAGGATTTCCTCTGGCAGGC<br>AACCTTATAATTTCA*CATATGAATATCCT*<br>*CCTTAG*	*avrA*	

For chromosomal gene insertion

1872FLAG-H2P2	GCCAGGAAGCGCTCGCCCCACACAAAAGCG<br>AGAGATGGGTTTTAATACGACTCACTATAGG<br>GCG	*grhD1*	
1872Rv+1s	CTGGGATCCCGCGGAAGACATGATTAAGGC	*grhD1*	

For gene cloning

HilDK6-F	GATACCATGGAAAATGTAACCTTTGTAA<br>GTAATAG	*hilD*	NcoI
HilDexR-PstI	TCCCTGCAGAACAATGATATTGAATAGC	*hilD*	PstI
PhoP-RV11	GAAGGATCCAGCACGACGCCGGCTGTC	*phoP*	BamHI
PhoP-FW22	ATCAAGCTTGCACCATAATCAACGCTAG<br>ACTG	*phoP*	HindIII
SirAFpMPMT3	CATAAGCTTCAAAAACGAGAGCAAAATCG	*sirA*	HindIII
SirARpMPMT3	TAAGGATCCGTACCGCCGGCGTCATAC	*sirA*	BamHI
1872Fw-K3	GAAGAGCTCGCACAAAAGGAGGATCAG	*grhD1*	SacI
1872Rv-K3	TATGGTACCTACTATCTGGAGCTGATTCTG	*grhD1*	KpnI
1872-SalIFw	GAAGTCGACGCACAAAAGGAGGATCAG	*grhD1*	SalI
1872Rv-3	CAGGGATCCAAAACCGTTTACTACGCTGGTC	*grhD1*	BamHI
HNS-NcoI	ACTACCATGGGCGAAGCACTTAAAATTC	*hns*	NcoI
Flag-His	TCACAAGCTTAGTGGTGGTGGTGGTGG<br>TGTTTATCGTCGTCATCTTTGTAGTCG	*hns*	HindIII
hns-Nco	CTACCATGGGCGAAGCACTTA	*hns*	NcoI
hns-22R	GCAATCTACAAGCTTTTATTGCTTGATC	*hns*	HindIII

For EMSAs

1872FW-1	CAGGTCGACAAGATCGCGCTCTGACCG	*grhD1*	SalI
1872RV-2	TGAGGATCCTGAGCTGATATCACTGGC	*grhD1*	BamHI
1872Rv-3	CAGGGATCCAAAACCGTTTACTACGCTGGTC	*grhD1*	BamHI
hilA1FBamHI	ATCGGATCCCTCTGAGAACTATTTGC	*hilA*	BamHI
hilA2RHindIII	GACAAGCTTTTCTGAGCGTAGCAGGG	*hilA*	HindIII
sigDBH1F	TCCCGACAGGATCCTTTTACCC	*sigD*	BamHI
sigDH3R	CGTTGTATAAGCTTTTTTGTAG	*sigD*	HindIII
stm1939 Fwd	ATCCAGAATCAGCTCC	*grhD1*	
stm1939 rv	CCGCCTCACCGATAAT	*grhD1*	
ges1	AATTTTGAATGTTCTAC	*ges*	
ges2	CAAGCGTATTCAGGCTGCACGGTTC	*ges*	
orgB PE 3	GCGTTTTACGCTTTATCAGTATCC	*orgB*	
PROM 2869	GCAAGGCTCCCTGCCT	*orgB*	
nucA FW	GGCAAGACGCGCAACTGG	*nucA*	
nuclease RV	CCGAAGGGCGCCG	*nucA*	
SsaBFBglII	GGCTAAGATCTTCGGCCCTGATATCCTG	*ssrAB*	BglII
SsrBRS6E	TTGGTCGACCGACAGATAGATGCCGG	*ssrAB*	SalI

Table S2. Primers used in this study. RE, restriction-enzyme for which a site was generated in the primer. Underlined letters indicate the respective restriction-enzyme site in the primer. The sequence corresponding to the template plasmids pKD4 or pSUB11 (Table S1) is in italic letters.

References

1. Hoiseth, S. K. & Stocker, B. Aromatic-dependent *Salmonella typhimurium* are non-virulent and effective as live vaccines. *Nature* **291**, 238-239 (1981).
2. Pérez-Morales, D. *et al.* The transcriptional regulator SsrB is involved in a molecular switch controlling virulence lifestyles of *Salmonella*. *PLoS Pathog* **13**, e1006497 (2017).
3. Bajaj, V., Lucas, R. L., Hwang, C. & Lee, C. A. Co-ordinate regulation of *Salmonella typhimurium* invasion genes by environmental and regulatory factors is mediated by control of *hilA* expression. *Mol Microbiol* **22**, 703-714, doi:10.1046/j.1365-2958.1996.d01-1718.x (1996).
4. Bustamante, V. H. *et al.* HilD-mediated transcriptional cross-talk between SPI-1 and SPI-2. *Proc Natl Acad Sci USA* **105**, 14591-14596 (2008).
5. Martínez, L. C. *et al.* Integration of a complex regulatory cascade involving the SirA/BarA and Csr global regulatory systems that controls expression of the *Salmonella* SPI-1 and SPI-2 virulence regulons through HilD. *Mol Microbiol* **80**, 1637-1656 (2011).
6. Martínez-Flores, I. *et al. In silico* clustering of *Salmonella* global gene expression data reveals novel genes co-regulated with the SPI-1 virulence genes through HilD. *Sci Rep* **6**, 37858 (2016).
7. Cano, D. A. *et al. Salmonella enterica* serovar Typhimurium response involved in attenuation of pathogen intracellular proliferation. *Infect Immun* **69**, 6463-6474, doi:Doi 10.1128/Iai.69.10.6463-6474.2001 (2001).
8. Ferenci, T. *et al.* Genomic sequencing reveals regulatory mutations and recombinational events in the widely used MC4100 lineage of *Escherichia coli* K-12. *J Bacteriol* **191**, 4025-4029 (2009).
9. Brosius, J. Plasmid vectors for the selection of promoters. *Gene* **27**, 151-160 (1984).
10. Datsenko, K. A. & Wanner, B. L. One-step inactivation of chromosomal genes in *Escherichia coli* K-12 using PCR products. *Proc Natl Acad Sci USA* **97**, 6640-6645 (2000).
11. Uzzau, S., Figueroa-Bossi, N., Rubino, S. & Bossi, L. Epitope tagging of chromosomal genes in *Salmonella*. *Proc Natl Acad Sci USA* **98**, 15264-15269 (2001).
12. Castelli, M. E., Véscovi, E. G. & Soncini, F. C. The phosphatase activity is the target for Mg2+ regulation of the sensor protein PhoQ in *Salmonella*. *J Biol Chem* **275**, 22948-22954 (2000).
13. Mayer, M. P. A new set of useful cloning and expression vectors derived from pBlueScript. *Gene* **163**, 41-46 (1995).
14. Husseiny, M. I. & Hensel, M. Rapid method for the construction of *Salmonella enterica* Serovar Typhimurium vaccine carrier strains. *Infect Immun* **73**, 1598-1605 (2005).
